# Concealed right upper lobe pulmonary venous variants on 3D-CTBA: morphological characterization and surgical implications

**DOI:** 10.3389/fmed.2026.1964027

**Published:** 2026-09-28

**Authors:** Luoyu Lian, Xin Luo, Dongliang Lin, Weiqing Zhang, Jinlong Huang, Shuchen Chen

**Affiliations:** 1 Department of Thoracic Surgery, Fujian Medical University Union Hospital, Fuzhou, China; 2 Department of Thoracic Surgery, Quanzhou First Hospital Affiliated to Fujian Medical University, Quanzhou, China; 3 Department of Cardiac and Thoracic Surgery, Longyan First Affiliated Hospital of Fujian Medical University, Longyan, China

**Keywords:** anatomical segmentectomy, bronchovascular anatomy, pulmonary venous variation, right top pulmonary vein, right upper lobe, thoracic surgery, three-dimensional reconstruction

## Abstract

**Introduction:**

The surgical anatomy of the right upper lobe pulmonary vein is variable and rare anomalies often go unnoticed in the first intraoperative exposure.

**Methods:**

3D-CTBA reconstructions were performed on 307 right-sided contrast-enhanced chest CT data with full segmentation of the arterial, venous and bronchial systems.

**Results:**

Anomalous right upper lobe vein anatomy was detected in 23 cases (7.49%) consisting of 18 right top pulmonary veins (RTPVs) and 5 cases of proposed cranially displaced central vein (CDCV). In all 18 RTPVs, a single venous trunk collecting S2 drainage followed a retrobronchial course posterior to the right main bronchus or bronchus intermedius. Thirteen drained directly into left atrium. The other five had translobar confluence with the inferior pulmonary vein system; one drained first into V6 before joining the inferior pulmonary vein. Surgically relevant arterial anomalies were concentrated in the RTPV subgroup. A central vein configuration was also identified, in which the venous trunk coursed in the interval between B1 and the adjacent roots of B2 and B3, consistently more cranial than expected. This pattern is called the cranially displaced central vein (CDCV).

**Discussion:**

Recognizing these precise anatomical configurations via preoperative 3D-CTBA is essential to avoid unexpected intraoperative hemorrhage during thoracic procedures.

## 1 Introduction

The surgical treatment of early-stage NSCLC has changed dramatically in the last decade. Due to the major randomised studies published in recent years, anatomic segmentectomy is now an accepted alternative to lobectomy for some peripheral NSCLC lesions of 2 cm or less (e.g., ground-glass opacity-positive tumors) (1, 2). Video assisted thoracic surgery (VATS) and robotic assisted thoracic surgery (RATS) segmentectomy are performed more and more frequently. In this setting, precise preoperative assessment of bronchovascular anatomy is essential. Safe segmentectomy relies on segmental and subsegmental vessels before division (3, 4).

Anatomic segmentectomy is performed in smaller and more variable intersegmental areas. Unexpected variations in the vascular system may cause bleeding problems, venous congestion, or infarction of the preserved lung, and difficulty maintaining adequate margin. The right upper lobe (RUL) is relevant here because of its complex bronchovascular structure. The classical description (5–7) of the venous drainage of RUL is divided into two groups: the anterior vein draining the apical and anterior lobe, and the central vein draining the posterior segment and coursing in the deep intersegmental plane relative to the B1, B2 and B3 bronchial roots. This is useful for routine planning but does not fully account for the variety of venous configurations present in modern 3D imaging (5, 6).

Larger 3D-CTBA has allowed small venous tributaries and spatial relation to the bronchial tree to be assessed more precisely than in previous cadaver or 2D imaging studies. This allowed better recognition of variants of the venous that may not be noticed by intraoperative exposure (8, 9). One such variant is the right top pulmonary vein (RTPV) which has anomalous posterior venous trunk collecting S2 drainage and flowing dorsally to the right main bronchus or bronchus intermedius and retrobronchial position makes surgical blind spots. Sometimes drainage into inferior pulmonary vein or V6 means that the RTPV is possible not only during right upper lobe resection, but also during lower lobe resection, and subcarinal dissections.

In addition to our 3D-CTBA database, we also found a different central vein. In the classical description, the central vein of the RUL runs between B2 and the roots of B1 and B3. The second one was different. The central vein of the RUL runs in between B1 and the roots of B2 and B3, consistently more cranially than expected. For the record, this position has not been described explicitly by the same criteria for the bronchial topology as previously described in previous thoracic imaging or surgical reports (5–7, 10). It may be useful for intersegmental dissection.

This work was therefore designed to (1) identify anomalous RUL venous configurations in a 3D-CTBA cohort; (2) describe anatomy, drainage patterns, and associated bronchial and arterial variations; (3) propose cranially displaced central vein (CDCV) for reproducible central venous position variant found in our series; and (4) discuss surgical relevance of the variants for right upper lobe resection, posterior segmentectomy, right lower lobe resection and subcarinal mediastinal dissection. Alongside its role as anatomical reference, the morphology reflects the development of computational tools in clinical practice. Workflows and surgical training increasingly incorporate artificial intelligence and large language modeling methods (11). Detailed preoperative morphological data may serve as a reliable baseline for future surgical navigation algorithms and AI-based planning systems.

## 2 Materials and methods

### 2.1 Study design and cohort definition

This single-center retrospective observational study was approved by the Institutional Review Board of Quanzhou First Hospital [Approval No. QY LUN (2022) 221]. Written informed consent was waived because the study involved retrospective analysis of anonymized imaging data and did not affect patient management.

To investigate the prevalence and anatomy of abnormal right upper lobe pulmonary veins, we retrospectively reviewed contrast enhanced chest CTs at Department of Thoracic Surgery Quanzhou First Hospital between October 2022 and October 2023. Three-dimensional reconstructions were obtained from all available data sets. 414 consecutive 3D models were first available. After excluding 107 left-sided models without right-sided bronchovascular reconstruction, 307 right-sided models were included in the final screening group consisting of 143 men and 164 women.

All 307 models were independently examined by 2 senior attending thoracic surgeons. Twenty-three RUL pulmonary vein cases were identified. The prevalence was 7.49% (23/307). According to definitions below, these cases were divided into right top pulmonary vein (RTPV) (*n* = 18, 5.86%) and cranially moved central vein (CDCV), *n* = 5, 1.63%), respectively. These 23 cases were the target group for the next analysis.

### 2.2 CT acquisition protocol and three-dimensional reconstruction

Contrast enhanced chest CT was performed using multidetector CT scanner using the standard thoracic enhanced scanning procedure at our institution. Image reconstruction was performed with slice thickness 0.625–1.0 mm and matrix of 512 × 512. Intravenous iodinated contrast medium was administered via power injector to provide adequate opacification of both arterial and vein to allow a reliable separation of arterial and vein.

Three-dimensional virtual bronchovascular models were modeled from the DICOM data using medical imaging software (Fudimai 3D Planning System; Chongqing Fudimai Digital Technology Co., Ltd., Chongqing, China). In each case, the tracheobronchial tree, pulmonary arterials, pulmonary veins and lung parenchyma were segmented separately to produce a 3-dimensional model. Small-caliber venous tributaries were considered at sub- and inter-segmental levels to detect anomalous vein pathways.

All reconstructions were independently re-assessed by 2 Thoracic surgeons who had experience in image-based preoperative planning for VATS and RATS anatomical lung resection. Each case was assessed independently before consensus classification. Disagreements with the anatomy were resolved by discussion. Reconstructed results were cross-referenced to the original axial and coronal contrast enhanced CT images before final classification. The work flow is summarized in Figure 1.

**Figure 1:**
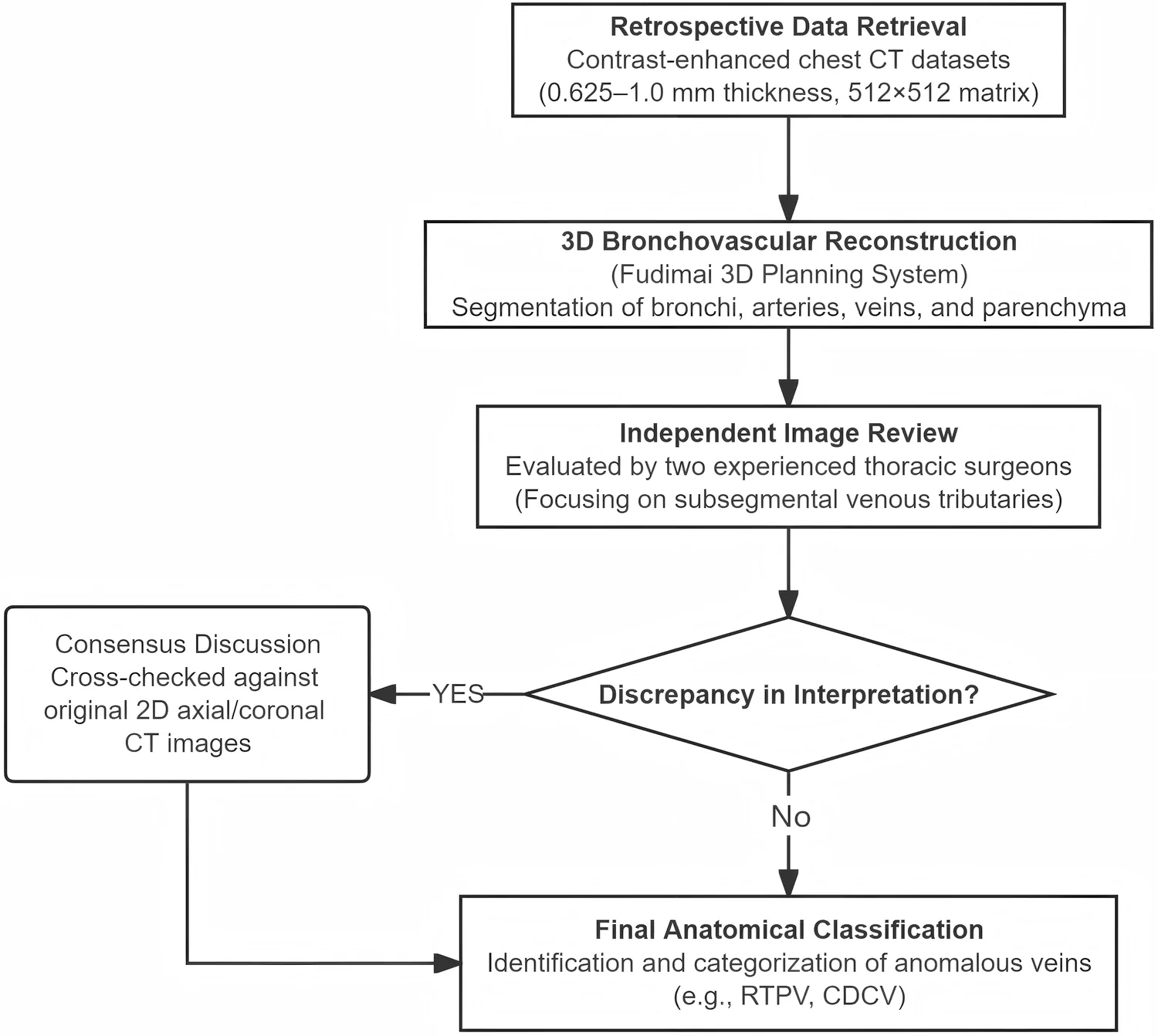
Study workflow for cohort selection, three-dimensional reconstruction, and anatomical classification. 414 3D models were screened from contrast enhanced chest CT examinations performed between October 2022 and October 2023. After excluding 107 left-sided models without complete right-sided bronchovascular reconstruction, 307 right-sided models were included. Two thoracic surgeons reconstructed the bronchi, pulmonary arteries and veins independently according to the original axial and coronal CT images. Disagreements were resolved by consensus. Twenty-three models with anomalous right upper lobe pulmonary veins were identified, consisting of 18 right top pulmonary veins and 5 cases of the proposed cranially displaced central vein (CDCV) pattern. CT, 3D-CTBA, 3D-CTBA, 3D computed tomography bronchography and angiogram.

### 2.3 CT acquisition protocol and three-dimensional reconstruction

The following anatomical and topological definitions were applied throughout the study to maintain consistency during image review.

#### 2.3.1 Pulmonary artery trunk classification

The superior trunk (Tr. sup) is the first independent branch arising from the right pulmonary artery mainstem. The inferior trunk (Tr. inf) is the second independent branch arising directly inferior or posteroinferior to Tr. sup. Trunk classification was made on the basis of independent ostia in the main pulmonary artery, not on the distal branching. Branching from the interlobar pulmonary artery or sharing a stem without independent mainstem origin are not considered separate trunks.

#### 2.3.2 Ascending and recurrent arterial branches

Ascending arteries are branches from the interlobar part of the right pulmonary arterial, passing through the fissure, and entering the upper lobe by the fissure. Each ascending branch is recorded according to the segment supplied. A recurrent artery is defined as S2-supplying branch from A1. An independently originating recurrent A2 branch is defined as S2-supplying branch from the anterior main pulmonary arterial trunk (Tr. sup) rather than a common stem with A1. This branch may occur unexpectedly during anterior hilar dissection.

#### 2.3.3 Definition of anomalous venous variants

Two anomalous venous configurations were defined.

Type A, the right top pulmonary vein (RTPV), was defined as an anomalous venous trunk draining the posterior segment (S2) and following a retrobronchial course dorsal to the right main bronchus or bronchus intermedius before reaching its terminal drainage site.

Type B (CDCV) is a central vein. The main trunk runs in the interval between B1 and B2 and B3 and travels cranial to B3 before joining the venous system. This is different from the central course of the central veins, where the trunk runs in the interval between B2 and B1 and B3 and joins the venous system caudally to B3.

#### 2.3.4 Terminal drainage pattern of the RTPV

For RTPV cases, the terminal drainage site was classified into three categories: (1) direct drainage into the left atrium; (2) drainage into the inferior pulmonary vein after crossing the interlobar plane; and (3) drainage into the superior segmental vein of the right lower lobe (V6).

#### 2.3.5 General classification of right upper lobe veins

To categorize the baseline venous anatomy, we applied the standard simplified vein models established by Shimizu et al. (6). Briefly: (1) Iab type: the anterior vein originates from both V1a and V1b; (2) Ib type: the anterior vein originates only from V1b; (3) Anterior type: the central vein is absent; and (4) Central type: the anterior vein is absent.

### 2.4 Statistical analysis

We used IBM SPSS Statistics, version 26.0 (IBM Corp. Armonk, NY, USA) primarily for descriptive anatomical studies. We used prevalence estimation and morphological analysis. Categorical variables are presented as counts and percentages. Cross-tabulation summarizes the co-occurrence of variants of vein with the associated bronchial and arterial branching patterns. No hypothesis testing was performed due to the small sample size and descriptive nature of the study.

## 3 Results

### 3.1 Baseline characteristics and overall prevalence

After 107 left-sided models were removed from the first set of 414 cases, 307 right-sided 3D-CTBA models were included in the study. The sample consisted of 143 men and 164 women. All included models were considered adequate for evaluation of subsegmental bronchovascular anatomy such as third-order bronchial branches, subsegmental arterial branches and intersegmental veins.

Out of 307 models tested 23 had anomalous right upper lobe pulmonary vein anatomy. In general prevalence was 7.49% (23/307). Eighteen were right top pulmonary vein (RTPV) cases representing 5.86% (18/307) of total cohort and 78.3% (18/23) anomalous cases. Five were cranially displaced central vein (CDCV), representing 1.63% (5/307) of total cohort and 21.7% (5/23) anomalous cases. These 23 cases were carried forward for analysis.

### 3.2 Concomitant bronchial and arterial variation patterns

In all 23 anomalous cases, the venous variant was accompanied by variation in adjacent bronchial branching and/or pulmonary arterial anatomy. Detailed anatomical characteristics for each case are summarized in Table 1.

**Table 1:** Anatomical characteristics of anomalous right upper lobe pulmonary venous variants (*n* = 23).

Variable	RTPV (*n* = 18)	CDCV (*n* = 5)	Total (*n* = 23)
Bronchial branching pattern, *n* (%)			
Typical pattern (B1/B2/B3)	6 (33.3)	5 (100.0)	11 (47.8)
Defective bifurcated pattern^a^	6 (33.3)	0 (0.0)	6 (26.1)
Complex/displaced pattern^b^	6 (33.3)	0 (0.0)	6 (26.1)
Main arterial trunk pattern, *n* (%)			
Tr. sup + A. pos	9 (50.0)	1 (20.0)	10 (43.5)
Tr. sup + A. ant	3 (16.7)	0 (0.0)	3 (13.0)
Tr. sup + A. pos + A. ant	2 (11.1)	2 (40.0)	4 (17.4)
Tr. sup + Tr. inf	2 (11.1)	0 (0.0)	2 (8.7)
Other complex patterns^c^	2 (11.1)	2 (40.0)	4 (17.4)
General venous classification, *n* (%)			
Anterior type	8 (44.4)	0 (0.0)	8 (34.8)
Iab type	7 (38.9)	0 (0.0)	7 (30.4)
Ib type	3 (16.7)	0 (0.0)	3 (13.0)
Central type (special variant)	0 (0.0)	5 (100.0)	5 (21.7)
High-risk & ectopic arteries, *n* (%)			
Absent	12 (66.7)	5 (100.0)	17 (73.9)
Independent recurrent A2 (RecA2_Indep)	5 (27.8)	0 (0.0)	5 (21.7)
Cross-segmental A2 from A1	1 (5.6)	0 (0.0)	1 (4.3)

RTPV, right top pulmonary vein; CDCV, cranially displaced central vein; Tr. sup, truncus superior; Tr. inf, truncus inferior; A. pos, posterior ascending artery; A. ant, anterior ascending artery.

a Defective bifurcated pattern includes B1-B2 + 3 (*n* = 3), B1 + 2-B3 (*n* = 2), and B1 + 3-B2 (*n* = 1).

b Complex/displaced pattern includes downward displacement of B2 to the bronchus intermedius (*n* = 2), B2 + BX1a-B3 + BX1b (*n* = 3), and B1 + BX2a-B3 + BX2b (*n* = 1).

c Other complex arterial patterns included isolated Tr. sup (*n* = 1) and Tr. sup + Tr. inf + A. pos (*n* = 1) in the RTPV group, and Tr. sup + Tr. inf + A. pos (*n* = 1) and Tr. sup + Tr. inf + A. pos + A. ant (*n* = 1) in the CDCV group.

Atypical bronchial branching was observed in 12 of 23 anomalous cases (52.2%). Bifurcation or complex branching was observed removing the usual trifurcation pattern of the right upper lobe bronchus. All 5 CDCV cases had the usual trifurcation pattern. Atypical bronchial branching was observed only in RTPV cases (66.7%). The different bronchial subtypes identified here are listed in Table 1 and their 3D shapes are summarized in Figures 2A–G. One anomalous case has high risk independently originating recurrent A2 branch from the anterior pulmonary arterial trunk (Figure 2H).

**Figure 2:**
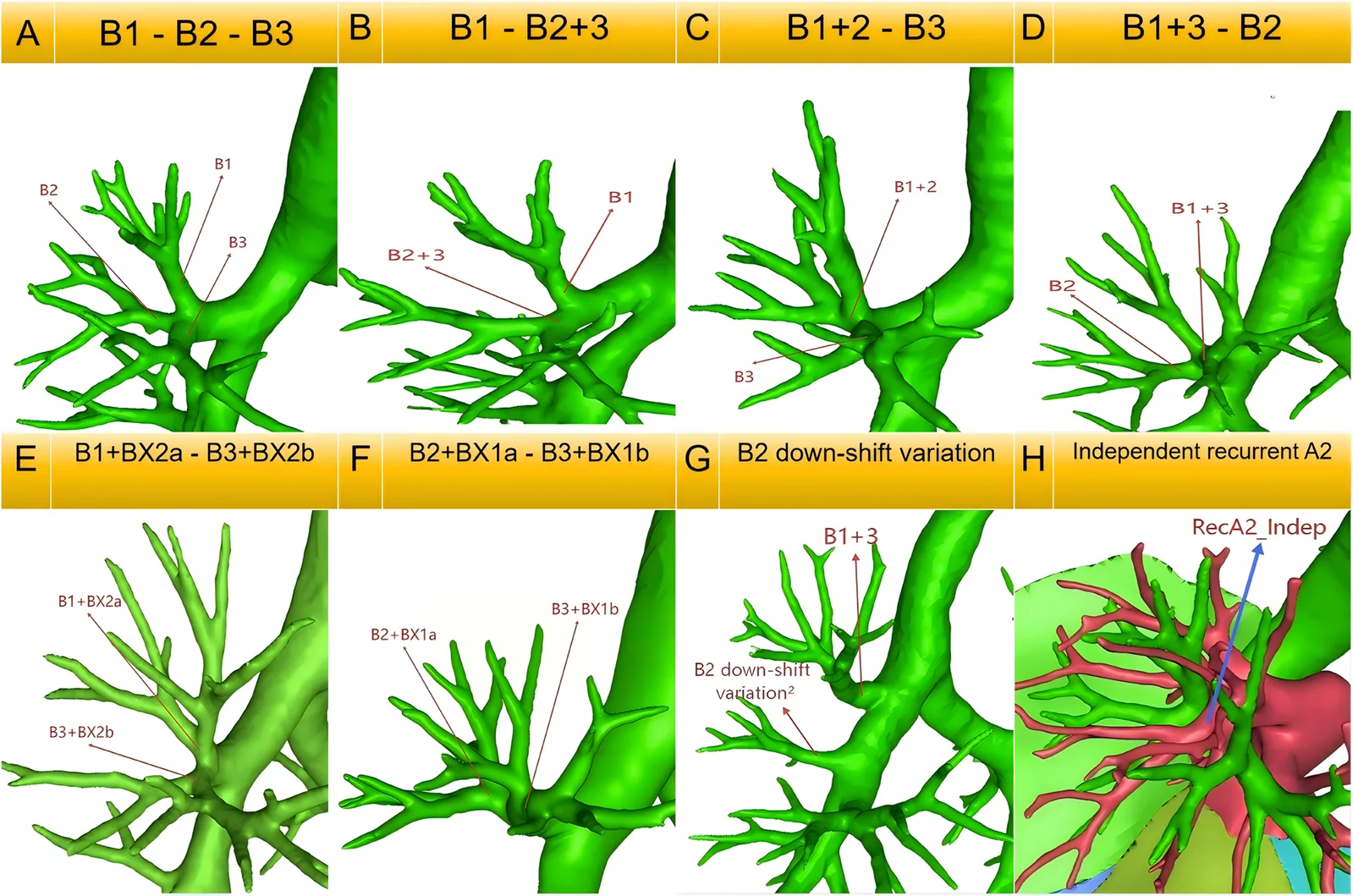
Bronchial branching patterns and associated arterial variations in anomalous RUL pulmonary venous cases. **(A)** Standard trifurcation pattern with independent B1, B2, and B3 branches. **(B)** B1–B2 + 3 bifurcation. **(C)** B1 + 2–B3 bifurcation. **(D)** B1 + 3–B2 bifurcation. **(E)** B1 + BX2a–B3 + BX2b branching pattern. **(F)** B2 + BX1a–B3 + BX1b branching pattern. **(G)** Caudal displacement of B2 toward the bronchus intermedius. **(H)** Representative case of independently originating recurrent A2 branch from anterior pulmonary arterial trunk. Bronchi and pulmonary vessels are colored according to color scheme shown in each panel: B1, apical segmental bronchus, B2, posterior segmental bronchus, B3, anterior segmental bronchus, BX, subsegmental bronchus displacement, A2, posterior segmental pulmonary artery.

Multiple pulmonary arterial branching patterns were found in the 23 cases. The most common configuration was Tr. sup + A. pos, found in 10 cases (43.5%), 9 RTPV cases and 1 CDCV case. Other configurations were Tr. sup + A. ant (3 cases 13.0%), Tr. sup + A. pos + A. ant (4 cases 17.4%), Tr. sup + Tr. inf (2 cases 8.7%), and other complex configurations (4 cases 17.4%), described in Table 1.

Six out of 23 anomalous cases (26%) had high risk ectopic branches. Five (21.7%) had independently originating recurrent A2 branch; one (4.3%) had transsegmental A2 branch in A1. All six cases were in the RTPV subgroup; no anomalous arterial branch was found in the CDCV subgroup; five of the six cases had atypical bronchial branching. This is not accidental. It shows a consistent clustering of the RTPV subset.

The associated bronchial and arterial variations in this series were primarily observed in RTPV patients. There was no control group, and no statistical analysis was done for the associations. Figure 3 shows schematic representations of the standard anterior vein, the typical central vein and two variants in this series: RTPV and CDCV.

**Figure 3:**
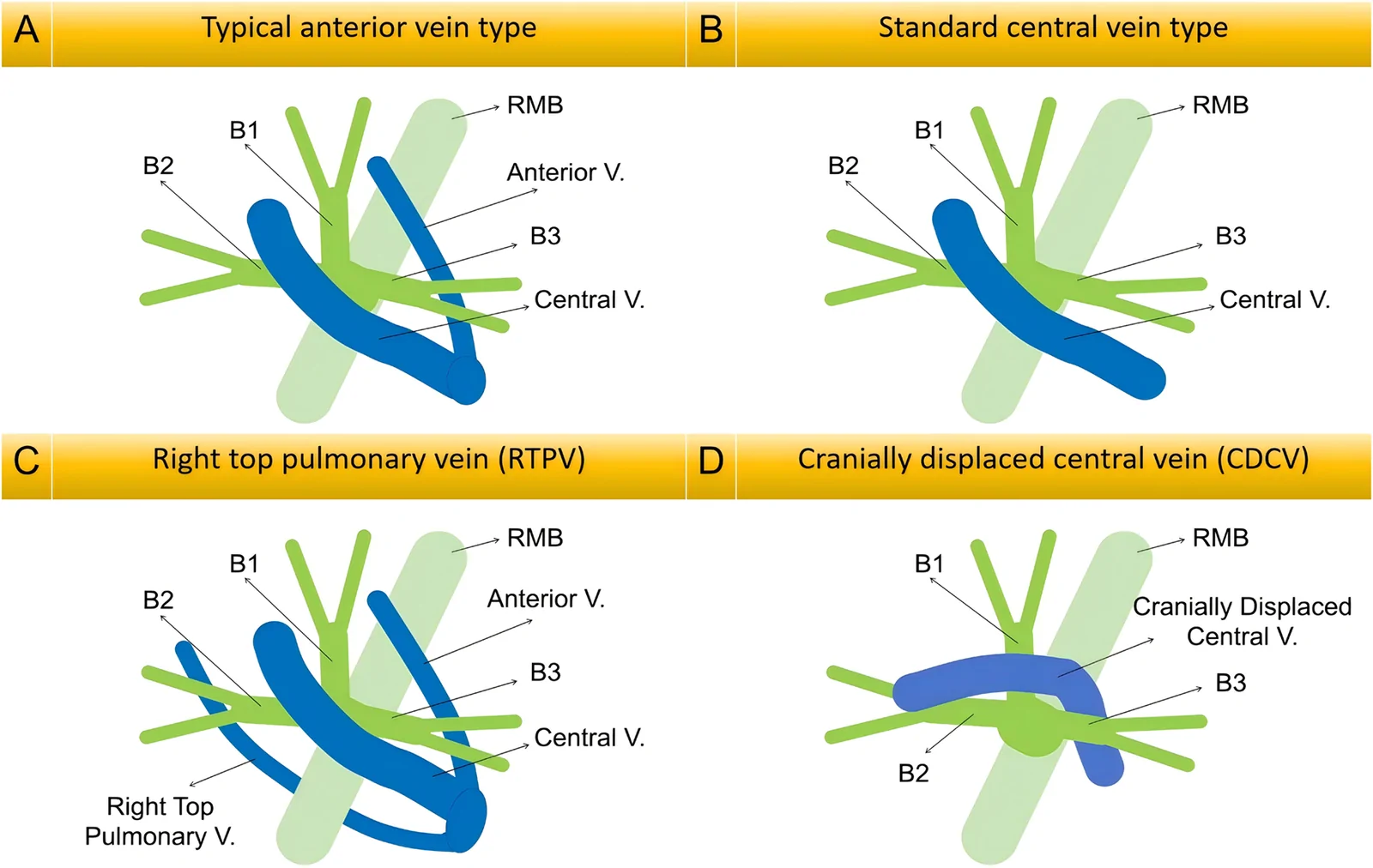
Schematic representations of standard and anomalous venous drainage patterns in the right upper lobe. **(A)** Anterior venous pattern, in which the principal venous trunk courses anterior to the right upper lobe bronchus. **(B)** Standard central venous pattern, in which the central vein follows a deep intersegmental course in relation to the independently branching B1, B2, and B3 bronchi and is positioned more caudally than the proposed CDCV pattern. **(C)** Right top pulmonary vein (RTPV), characterized by a retrobronchial course dorsal to the right main bronchus or bronchus intermedius. **(D)** CDCV pattern of central veins with more cranial course between B1 and adjacent independent branching root B2 and B3. B1, apical segmental bronchus; B2, posterior segmental bronchus; B3, anterior segmental bronchus; CDCV pattern of central vein; RTPV right top pulmonary vein.

### 3.3 Anatomical topography and terminal drainage of RTPV

The anatomy of the 18 RTPV cases is summarized in Table 2. Retrobronchial course is found in all cases. In each case, the anomalous vein trunk moves dorsally towards the right main bronchus (bronchus intermedius) before reaching its end drainage site. Figure 4 shows anterior and posterior views of this retrobronchial course.

**Table 2:** Terminal drainage patterns and associated arterial variations in RTPV (*n* = 18).

Terminal drainage Site	*n* (%)	Venous subtype distribution	High-risk arterial anomalies
Left atrium (LA)	13 (72.2)	Iab (*n* = 6), Anterior (*n* = 4), Ib (*n* = 3)	RecA2_Indep (*n* = 3), Cross-segmental A2 from A1 (*n* = 1)
Inferior pulmonary vein (IPV)	4 (22.2)	Anterior (*n* = 3), Iab (*n* = 1)	RecA2_Indep (*n* = 2)
Segmental vein V6	1 (5.6)	Anterior (*n* = 1)	None

RTPV, right top pulmonary vein; LA, left atrium; IPV, inferior pulmonary vein; RecA2_Indep, independently originating recurrent arterial branch to segment 2.

RTPVs draining into the IPV or V6 accounted for 27.8% (5/18) of RTPV cases.

**Figure 4:**
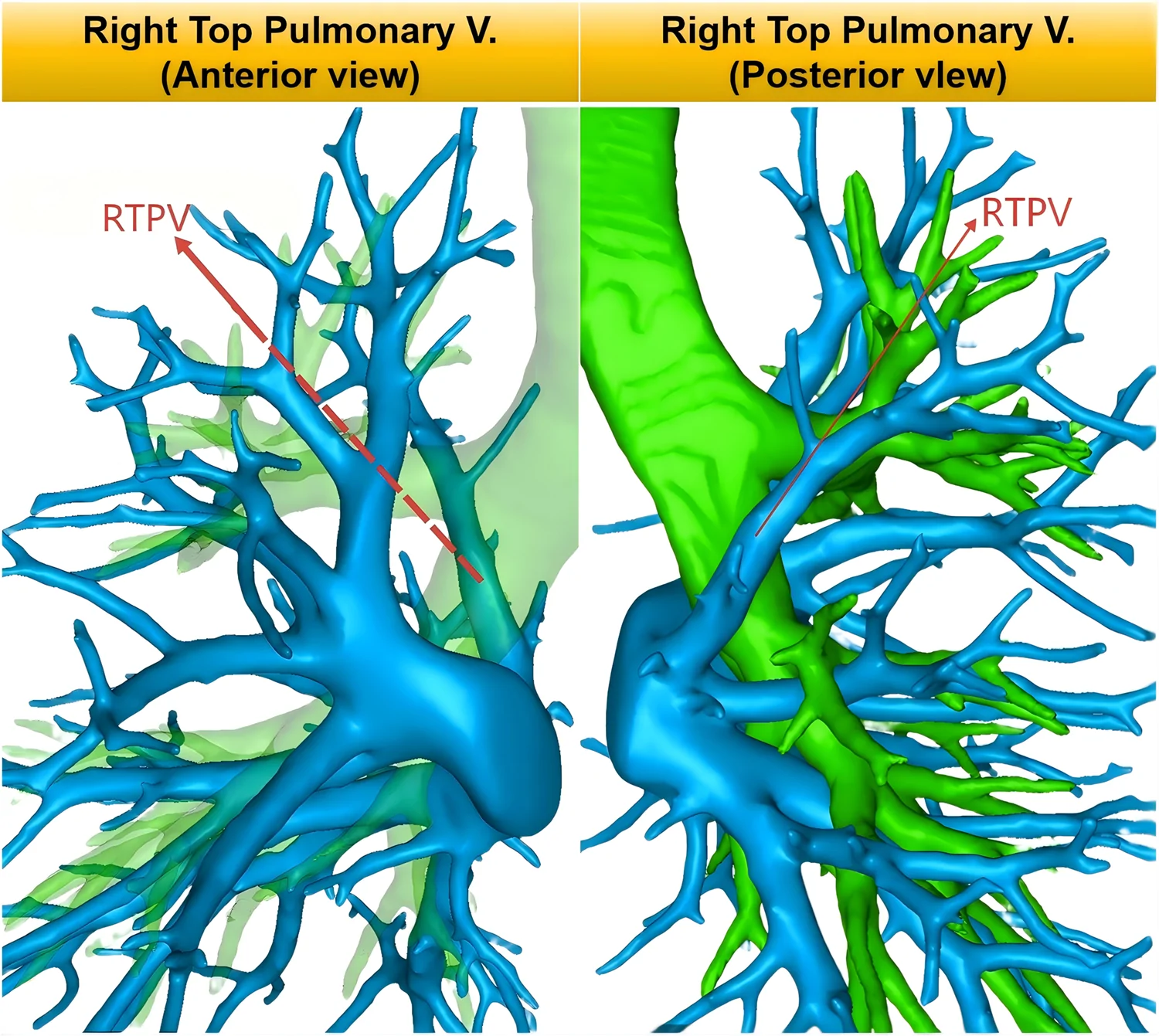
Representative three-dimensional images showing the retrobronchial course of the right top pulmonary vein. **(A)** Anterior view showing the relationship of the anomalous venous trunk to the right upper lobe bronchus and pulmonary hilar structures. **(B)** Posterior view showing the anomalous vein passing dorsal to the right main bronchus or right mucosus, before reaching the drainage site at the end. RTPV (Right top pulmonary vein) arrows. Color codes of the bronchi, pulmonary arteries and pulmonary veins (BI, bronchus intermedius); RMB, right main bronchus; RTPV, right top pulmonary vein).

With respect to terminal drainage, 13 of the 18 RTPV cases (72.2%) drained directly into the left atrium. In these cases, the anomalous vein entered the superior or posterior aspect of the left atrium without crossing the interlobar fissure.

The remaining 5 RTPV cases (27.8%) had translobar drainage patterns. Four (22.2%) drained into the inferior pulmonary vein after crossing the interlobar plane and one (5.6%) into the superior segmental vein of the right lower lobe (V6). These translobar drainage patterns are particularly important because the anomalous vein may be mistakenly interpreted as a lower lobe vein branch during right lower lobectomy or S6 segmentectomy. Examples of RTPV draining into left atrium, inferior pulmonary vein and V6 are shown in Figure 5.

**Figure 5:**
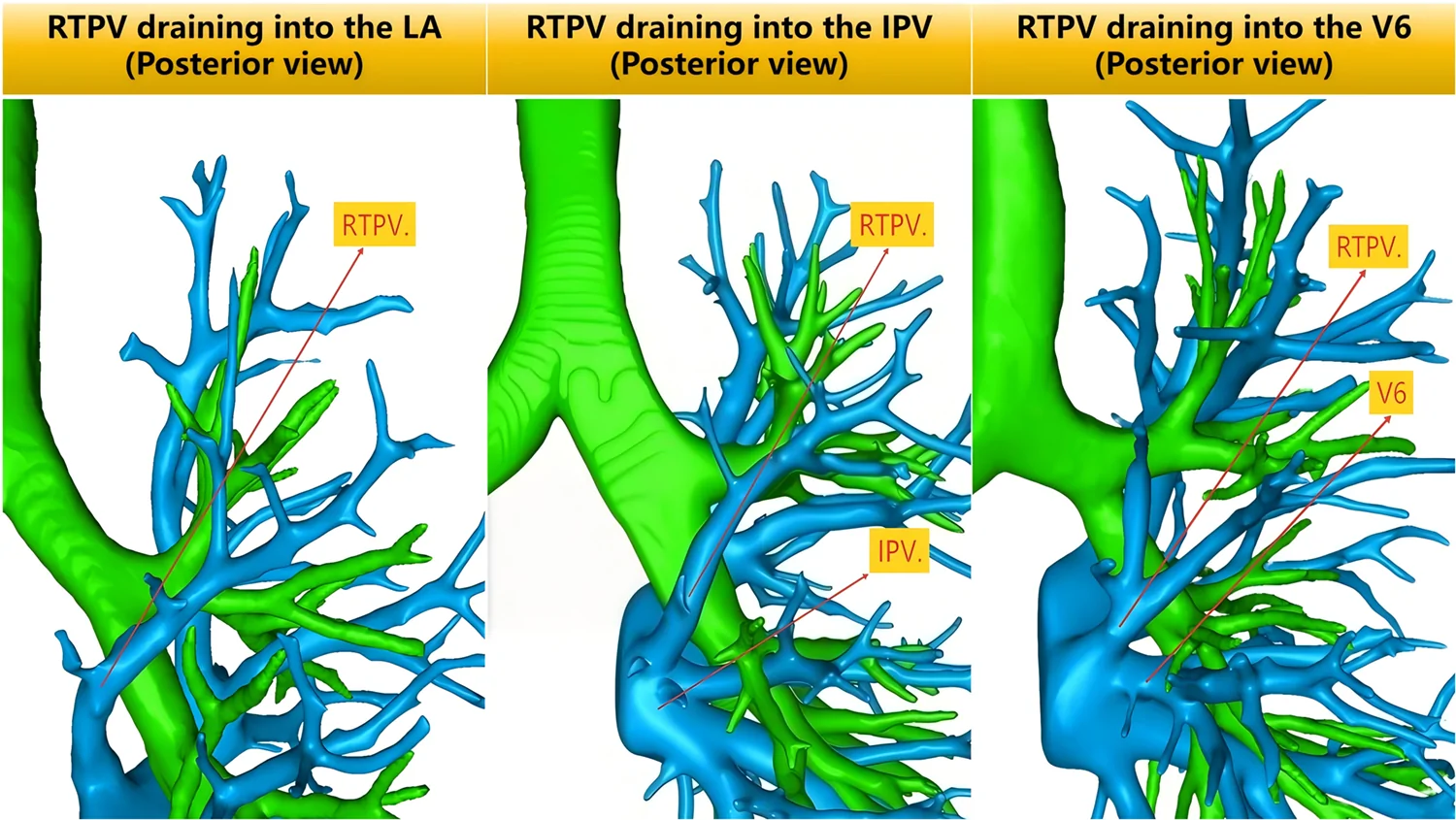
Representative terminal drainage patterns of the right top pulmonary vein. **(A)** RTPV draining directly into the left atrium. **(B)** RTPV crossing the interlobar plane and joining the inferior pulmonary vein. **(C)** RTPV joining the superior segmental vein of the right lower lobe (V6). Arrows represent the anomalous vein trunk and its confluence. LA = left atrium; IPV = inferior pulmonary vein; RTPV = right top pulmonary vein; V6 = superior segmental vein of the right lower lobe.

In the 18 RTPV cases, the vein type distribution was as follows: anterior type 8 cases (44,4%), Iab type 7 cases (38,9%) and Ib type 3 cases (16,7%). High risk ectopic arterial branches were found in 6 RTPV cases, 5 with independent recurrent A2 branch and 1 with cross-segmental A2 branch A1 (Table 2).

### 3.4 Morphological features of CDCV

Five CDCV cases were identified, a frequency of 1.63% (5/307) in all cases. In all 5 cases, the posterior V2a, V2b, and V2c joined the anterior tributary V3a to form a common venous trunk. The most distinctive feature of this pattern was the cranial position of the trunk in relation to the segmental bronchial roots. In other words, the venous trunk travelled between B1 and B2 and B3, while B1, B2 and B3 emerged independently. This positional relationship was observed in all 5 CDCV cases.

All CDCV cases showed the central vein pattern in the general vein classification and retained the typical bronchial trifurcation pattern. No high-risk ectopic arterial branch was found in this subgroup (Table 1). The displaced vein trunk may be mistaken intraoperatively for a different intersegmental vein if not located before surgery. Schematic and 3-D reconstructions of CDCV are shown in Figure 6.

**Figure 6:**
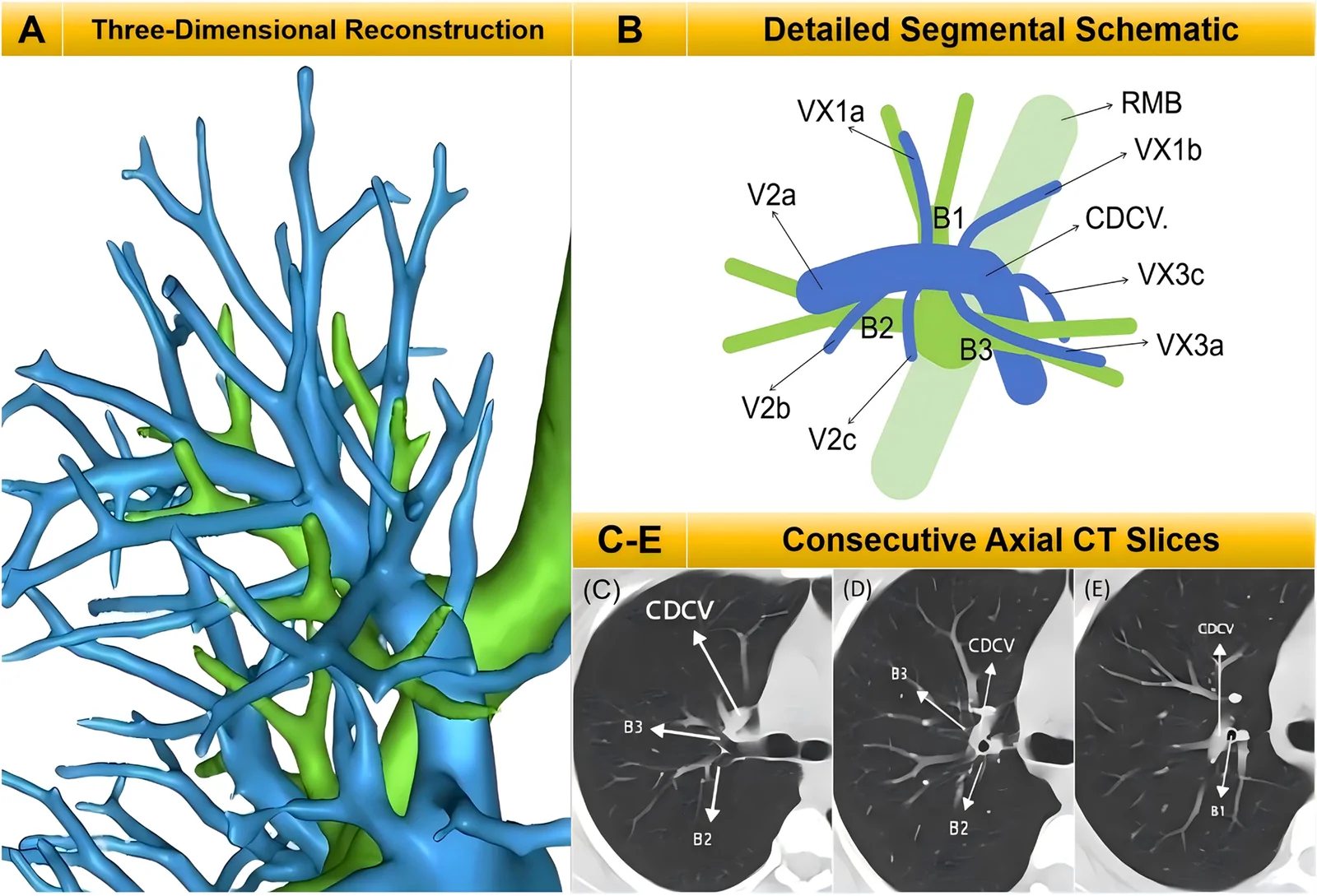
Representative three-dimensional reconstruction and corresponding axial CT images of the proposed CDCV pattern. **(A)** Three-dimensional bronchovascular reconstruction of the proposed CDCV trunk in red. The venous trunk follows a cranially displaced course between B1 and the adjacent independent branching roots B2 and B3. **(B)** Schematic representation of the segmental and subsegmental vein anatomy. The posterior veins V2a, V2b and V2c join with the anterior vein V3a informing the CDCV trunk. **(C–E)** Repeated thin section CT images in cranial-to-caudal sequence. Red arrows indicate the course of the venous trunk relative to B1, B2 and B3 bronchial roots. These images correspond to the spatial relationship seen on the 3D reconstruction. B1, apical segmental bronchus; B2, posterior segmental bronchus; B3, anterior segmental bronchus; CDCV, cranially displaced central venous pattern; CT (computed tomography). V2a – c posterior segmental venous tributaries; V3a anterior segmental venous tributary).

## 4 Discussion

In this review of three-dimensional CT models, abnormal right upper lobe pulmonary venous anatomy was observed in 7.49% of the right-sided models. The prevalence of RTPV was 5.86%, consistent with the figures reported in (12). Two important results were found. First, the RTPV was composed of a venous trunk running dorsal to the right main bronchus or bronchus intermedius before joining the venous system and a large proportion of cases draining into the inferior vein rather than right superior vein, which must be identified before any right-sided lobar procedure. Second, a central venous configuration was identified (the cranially displaced central vein) in which principal trunk moved in the interval between B1 and B2 and cranial to B3 before joining the venous system, whereas typical central vein runs between B2 and the roots of B1 and B3 and joins the venous system caudal to B3. All five CDCV cases showed a standard B1, B2, B3 trifurcation pattern.

The surgical implications are direct. Both configurations are difficult to predict during routine exposure. The RTPV is inserted entirely posterior to the right main bronchus, unlike anterior hilar dissection. An unprepared surgeon may assume that the space is empty. The CDCV is moved far from the expected location, and is more likely to get injured in segmentectomy (especially S2 resection).

This result extends the general description of right upper lobe pulmonary veins. Variations are not limited to differences in confluence patterns. Arterial space-course and bronchial topology differ too, and often together. Anomalous right upper lobe vein patterns should be viewed as part of a complex variation of the bronchovascular complex. In our series, RTPV is often associated with abnormal branching of the arterial and bronchial lobes, as observed in other studies (8, 13). We designated the independently originating recurrent A2 branch (RecA2_Indep) as ‘high-risk’ due to its specific vulnerability during anterior hilar dissection. In standard anatomy, the recurrent A2 shares a common stem with A1. When RecA2 originates independently from the anterior mainstem, an operator might divide the first identified arterial trunk and mistakenly assume the anterior arterial supply is fully controlled. Furthermore, its ectopic course frequently places it deep and medial to the anomalous venous trunk. If not pre-identified, this independent artery is highly susceptible to inadvertent transection during venous isolation in S2 segmentectomy. The surgical hazards of such ectopic recurrent arteries have been corroborated by recent anatomical studies (8, 13). Safe segmentectomy depends on the anatomical identification before any structure is split. Preoperative 3D CTBA recognition of the patterns directly reduces the risk.

Classical anatomical descriptions established the basis for right upper lobe bronchovascular organization and continue to guide intraoperative dissection (7, 14, 15). These models were derived from cadaveric dissection and two-dimensional imaging but did not fully capture the complex 3-dimensional relationships between the vascular and bronchial structures. As anatomic segmentectomy occurs more often, simplified anatomical templates become practical limitations.

Prior reports have characterized the RTPV as a rare but important blood vessel anomaly, known as retrobronchial vein and occasionally drainage into inferior vein or directly into left atrium (16–18). We reproduce these reports with additional details of bronchial and arterial branching patterns in the RTPV subgroup. Identifying these variations provides preoperative planning with a more complete basis.

The CDCV pattern has not been defined explicitly as a distinct anatomical form in the literature (5, 6). Five cases were identified (5/307). The low prevalence may explain why it was not reported in earlier systematic reports. All five cases were standard trifurcation bronchial pattern. No high risk arterial variants were observed. The small number of cases makes interpretation difficult. However, the same anatomical structure was found in all five cases.

This paper supports a wider role of 3D reconstruction in thoracic surgery. Previous work showed that 3D reconstruction improves the operating surgeon's knowledge of patient-specific pulmonary anatomy and improves the safety of segmentectomy (3, 4, 9). Our data add to this evidence. The ability of 3D-CTBA to map anomalous veins and their spatial relationship with bronchial and arterial systems is shown here and is actionable—for both right upper lobe and right lower lobe resection planning.

These anatomical variants carry specific operative consequences that are best understood in context.

### 4.1 RTPV

The RTPV drains S2 but travels posterior to the right main bronchus or bronchus intermedius and discharges into the left atrium or left lower lobe. Four scenarios are worth considering: First, when the lower lobe is resectioned, the RTPV is divided, so the upper lobe loses its S2 flow. Congestion or infarction of the upper lobe follows. Second, when the upper lobe is resection is retrobronchial, and the RTPV is retrobronchial and not on the standard anterior hilar plane. The surgeon who uses classical anatomy will not expect a vessel there, and bleeding is difficult. Third, subcarinal lymph nodes at station 7 expose the same retrobronchial corridor. An unrecognized RTPV can be torn during nodal clearance. Fourth, S2 segmentectomy requires identification of the correct intersegmental plane. If the RTPV has not been mapped preoperatively, its anomalous course impedes standard plane development and compromises the margin of resection (19). Preoperative identification of RTPV and its trajectory is therefore mandatory before any of these procedures.

### 4.2 CDCV and S2 segmentectomy

RTPV and CDCV patterns affect the surgical anatomy of S2. CDCV is disorienting intraoperatively. Rather than joining the veins at the superficial fissural surface caudal to B3, the central trunk of CDCV passes cranial to B3 and enters the system from a deeper deeper position. Unlike orientation, the deeper position of the trunk makes the dissection more hilar. Surgical trauma increases. In both cases, preoperative 3D CTBA mapping of the venous course allows the surgeon to predict what the surgeon will encounter before the first dissection is performed.

The implications of the RTPV beyond lung surgery. Its course posterior to the right main bronchus or bronchus intermedius places it directly in the subcarinal and posterior mediastinal dissection planes used for esophageal surgery. This is not a theoretical problem. Mediastinoscopic and minimally invasive esophageal surgery is increasingly performed and the field of operation is restricted. A unrecognized RTPV crossing this space creates hemorrhagic risk in a fast vascular control is difficult (20–22).

In summary, the RTPV and CDCV patterns have operative consequences that extend across right upper lobectomy, posterior segmentectomy, subcarinal nodal dissection, and posterior mediastinal dissection. None of these procedures should proceed without preoperative 3D-CTBA review when either variant is suspected or the anatomy appears atypical on standard axial imaging.

The embryological basis of these venous variants cannot be established from imaging data alone. The morphological patterns observed here, however, suggest that these are structured developmental variants rather than random anatomical anomalies.

The most plausible explanation for the CDCV pattern is aberrant integration of the intersegmental venous plexus to form the central venous trunk. Remodeling of this plexus at a relatively cranial level may redirect the final trunk to a higher position in the lobe. This is speculation. It cannot be confirmed from imaging alone, but it is consistent with the stable position pattern observed in all five cases. To avoid conceptual confusion, it is necessary to define the CDCV as an independent anatomical subtype rather than a subtle positional deviation. Anatomically, the CDCV demonstrates a discrete topological shift across the bronchial hierarchy—moving explicitly from the conventional B2/B1 + B3 plane to the higher B1/B2 + B3 interval—while maintaining a standard trifurcate bronchial architecture. Embryologically, this consistent pattern across multiple cases suggests a determinate developmental event, specifically the anomalous cranial integration of the intersegmental venous plexus, rather than a random anatomical variation. Establishing the CDCV as a distinct entity provides clinicians with a precise structural framework to anticipate its deeper, atypical course during intersegmental dissection.

For RTPV, one would think incomplete remodeling of a primitive posterior vein channel toward the standard anterior hilar drainage, hence the retrobronchial course of the anomalous trunk, or variable end drainage (left atrium, inferior pulmonary vein, or V6) depending on remodeling arrest extent. These developmental interpretations remain speculative. They should be regarded as anatomical hypotheses, not established mechanisms.

The present study has several strengths. The analysis was based on a consecutive institutional 3D-CTBA database. Explicit anatomical definitions were applied throughout. Venous anomalies were assessed together with associated bronchial and arterial patterns rather than in isolation. The emphasis on operative implications adds direct relevance for practicing thoracic surgeons.

There are some limitations: First, we are in a single-center retrospective imaging study. The prevalence and variations described may not fully reflect the other populations. Second, there are very few anomalous cases, especially for the CDCV subgroup. The morphological consistency is found for all five CDCV cases but true prevalence and variations need to be validated in multicenter cohorts with larger sample sizes. Third, our study carries an inherent selection bias. We exclusively analyzed patients who underwent contrast-enhanced CT, naturally excluding those unable to tolerate contrast agents due to severe allergies or renal insufficiency. Fourth, the accuracy of 3D-CTBA is not absolute. Physically, it is constrained by the spatial resolution of the original CT scans (0.625–1.0 mm slice thickness), making it difficult to capture extremely tiny subsegmental veins. Practically, the reconstruction quality relies heavily on the anatomical expertise of the software operators. To minimize human error, two experienced thoracic surgeons independently verified all models, and complex variants were adjudicated through multi-person consensus. Despite these strict cross-checks, we cannot guarantee 100% accuracy for every minute branch. In future workflows, integrating machine learning algorithms (23) and advanced computational visualization (24) may help standardize this process and improve precision. Fifth, the proposed embryological explanations remain speculative, as they are based on morphological inference rather than direct developmental evidence.

## 5 Conclusions

In 307 right 3D-CTBA models 23 right upper lobe pulmonary venous variation occurred. The RTPV had a retrobronchial course with translobar confluence into the inferior pulmonary venous system in 5 cases. Remarkably reproducible but uncommon central venous configuration with trunk cranial to the B3 root. Further studies will be needed to confirm the proposed CDCV pattern, verify its reproducibility and define its clinical significance. Preoperative 3D-CTBA may help identify patient specific venous anatomy before right upper or right lower lobe procedures.

## Data Availability

The original contributions presented in the study are included in the article/Supplementary Material, further inquiries can be directed to the corresponding authors.

## References

[1] Altorki N Wang X Kozono D Watt C Landrenau R Wigle D. Lobar or sublobar resection for peripheral stage IA non–small-cell lung cancer. N Engl J Med. (2023) 388:489–98. 10.1056/NEJMoa2212083 36780674 PMC10036605

[2] Saji H Okada M Tsuboi M Nakajima R Suzuki K Aokage K. Segmentectomy versus lobectomy in small-sized peripheral non-small-cell lung cancer (JCOG0802/WJOG4607L): a multicentre, open-label, phase 3, randomised, controlled, non-inferiority trial. Lancet. (2022) 399:1607–17. 10.1016/S0140-6736(21)02333-3 35461558

[3] Zhang M Liu D Wu W Zhang H Mao N. Preoperative 3D-CT bronchography and angiography facilitates single-direction uniportal thoracoscopic anatomic lobectomy. Ann Transl Med. (2019) 7:526. 10.21037/atm.2019.09.135 31807508 PMC6861819

[4] Wu W-B Xu X-F Wen W Xu J Zhu Q Pan X-L. Three-dimensional computed tomography bronchography and angiography in the preoperative evaluation of thoracoscopic segmentectomy and subsegmentectomy. J Thorac Dis. (2016) 8:S710–5. 10.21037/jtd.2016.09.43 28066674 PMC5179351

[5] Nagashima T Shimizu K Ohtaki Y Obayashi K Kakegawa S Nakazawa S. An analysis of variations in the bronchovascular pattern of the right upper lobe using three-dimensional CT angiography and bronchography. Gen Thorac Cardiovasc Surg. (2015) 63:354–60. 10.1007/s11748-015-0531-1 25724170 PMC4454828

[6] Shimizu K Nagashima T Ohtaki Y Obayashi K Nakazawa S Kamiyoshihara M. Analysis of the variation pattern in right upper pulmonary veins and establishment of simplified vein models for anatomical segmentectomy. Gen Thorac Cardiovasc Surg. (2016) 64:604–11. 10.1007/s11748-016-0686-4 27435809 PMC5035324

[7] Boyden EA Scannell JG. An analysis of variations in the bronchovascular pattern of the right upper lobe of 50 lungs. Am J Anat. (1948) 82:27–73. 10.1002/aja.1000820103 18919562

[8] Li Z Kong Y Wu W Chen S Zhang X. What is the correlation between the defective and splitting posterior segmental bronchus and recurrent artery crossing intersegmental planes in the right upper lobe? Front Surg. (2023) 10:1113783. 10.3389/fsurg.2023.1113783 36860942 PMC9968833

[9] Chen Z-H Chu X-P Zhang J-T Fu R Kang J Chen J-H. The regularity of anatomical variations of dominant pulmonary segments in the right upper lobe. Thorac Cancer. (2023) 14:462–9. 10.1111/1759-7714.14763 36511202 PMC9925340

[10] Wang J Lin H Bian C Chen Z Huang J Xia Y. A modified system for classifying the bilateral superior pulmonary veins using three-dimensional computed tomography bronchography and angiography images. J Thorac Dis. (2021) 13:5933–41. 10.21037/jtd-21-985 34795941 PMC8575819

[11] Lian L Luo X Chipusu K Ashraf MA Wong KKL Zhang W. Large language models evaluation of medical licensing examination using GPT−4.0, ERNIE Bot 4.0, and GPT−4o. Bioengineering. (2026) 13:113. 10.3390/bioengineering13010113 41596044 PMC12837214

[12] Shiina N Kaga K Hida Y Sasaki T Hirano S Matsui Y. Variations of pulmonary vein drainage critical for lung resection assessed by three-dimensional computed tomography angiography. Thorac Cancer. (2018) 9:584–8. 10.1111/1759-7714.12621 29573182 PMC5928364

[13] Li Z Kong Y Li B Lv W Zhang X. The detailed classification of the interlobar artery and the artery crossing intersegmental planes in the right upper lobe. Front Oncol. (2023) 13. 10.3389/fonc.2023.1195726 PMC10225715 37256176

[14] Nakazawa S Shimizu K Kawatani N Obayashi K Ohtaki Y Nagashima T. Right upper lobe segmentectomy guided by simplified anatomic models. JTCVS Techniques. (2020) 4:288–97. 10.1016/j.xjtc.2020.08.018 34318050 PMC8304484

[15] Yamashita H. Roentgenologic Anatomy of the Lung. Tokyo: Igaku-Shoin (1978).

[16] Asai K Urabe N Yajima K Suzuki K Kazui T. Right upper lobe venous drainage posterior to the bronchus intermedius: preoperative identification by computed tomography. Ann Thorac Surg. (2005) 79:1866–71. 10.1016/j.athoracsur.2004.12.014 15919274

[17] Miyamoto N Yoshida M Takashima M Matsumoto D Kawakita N Tsuboi M. Classifying the destination of right top pulmonary vein in 31 clinical cases. Gen Thorac Cardiovasc Surg. (2021) 69:1192–5. 10.1007/s11748-021-01604-6 33586095

[18] Akiba T Morikawa T Inagaki T Nakada T Ohki T. A new classification for right top pulmonary vein. Ann Thorac Surg. (2013) 95:1227–30. 10.1016/j.athoracsur.2013.01.011 23434252

[19] Polaczek M Szaro P Jakubowska L Zych J Religioni J Orlowski TM. Pulmonary veins variations with potential impact in thoracic surgery: a computed-tomography-based atlas. J Thorac Dis. (2020) 12:383–93. 10.21037/jtd.2020.01.34 32274104 PMC7139082

[20] Sato Y Tanaka Y Ohno S Endo M Okumura N Matsuhashi N. Right top pulmonary vein is a venous anomaly of which surgeons should be aware in subcarinal dissection for thoracoscopic esophagectomy: a case report and literature review. World J Surg Oncol. (2022) 20:160. 10.1186/s12957-022-02635-w 35590376 PMC9121563

[21] Matsubara T Hirahara N Zotani H Tabara N Tabara H Tajima Y. Three-dimensional computed tomography image-oriented successful thoracoscopic subtotal esophagectomy for an esophageal cancer patient with an anomalous right superior pulmonary vein. Int J Surg Case Rep. (2020) 76:178–82. 10.1016/j.ijscr.2020.09.196 33038843 PMC7550825

[22] Onodera Y Taniyama Y Sakurai T Hikage M Sato C Takaya K. Thoracoscopic esophagectomy with subcarinal lymph node dissection in the prone position for esophageal cancer with a right superior pulmonary vein anomaly: a case report. Surg Case Rep. (2019) 5:6. 10.1186/s40792-019-0567-z 30644002 PMC6331349

[23] Wong KKL Zhang A Yang K Wu S Ghista DN. GCW-UNet segmentation of cardiac magnetic resonance images for evaluation of left atrial enlargement. Comput Methods Programs Biomed. (2022) 221:106915. 10.1016/j.cmpb.2022.106915 35653942

[24] Yang Y Liu X Xia Y Liu X Wu W Xiong H. Impact of spatial characteristics in the left stenotic coronary artery on the hemodynamics and visualization of 3d replica models. Sci Rep. (2017) 7:15452. 10.1038/s41598-017-15620-1 29133915 PMC5684364

