# Supplementary material for: Concealed right upper lobe pulmonary venous variants on 3D-CTBA: morphological characterization and surgical implications
Source: Front Med (Lausanne). 2026 Sep 28;13:1964027. doi: 10.3389/fmed.2026.1964027 (PMC13655120; doi:10.3389/fmed.2026.1964027)

NO. 1

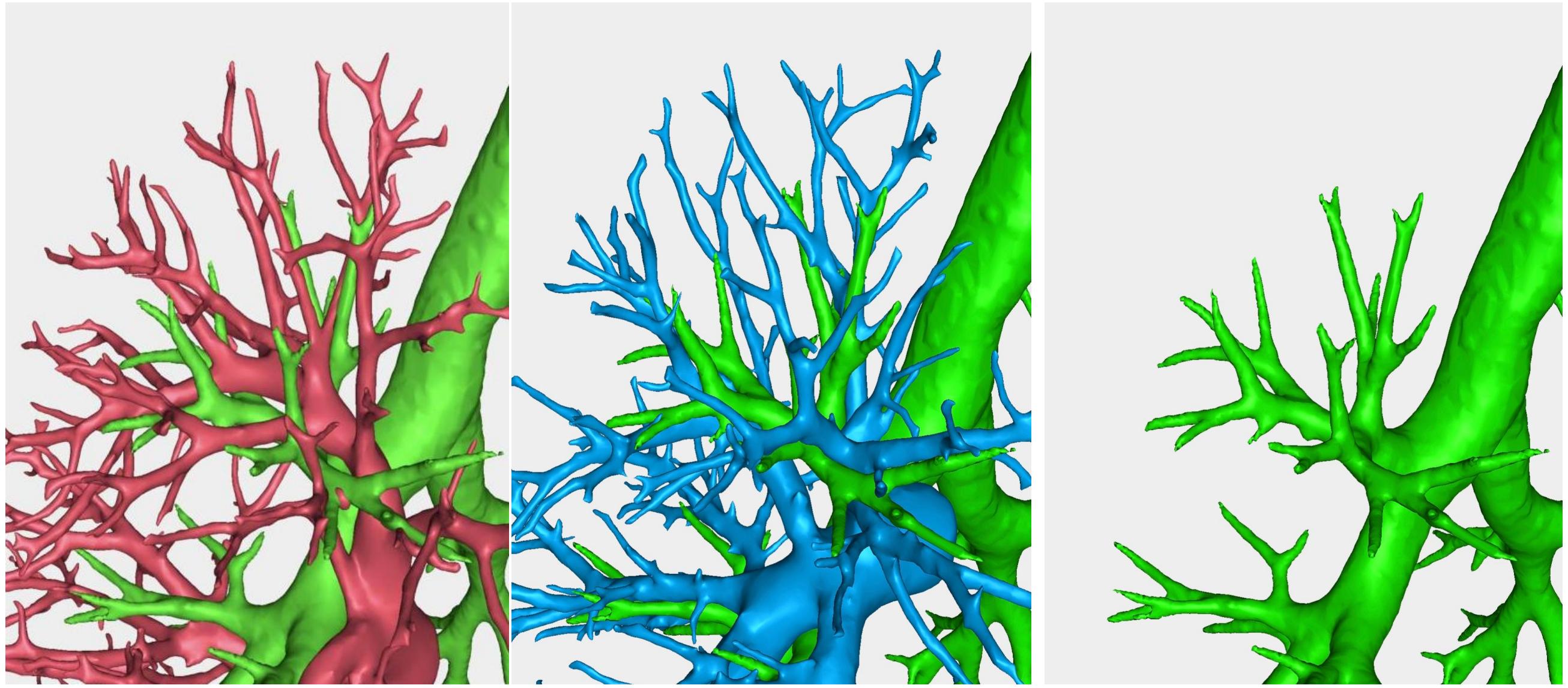

No. 2

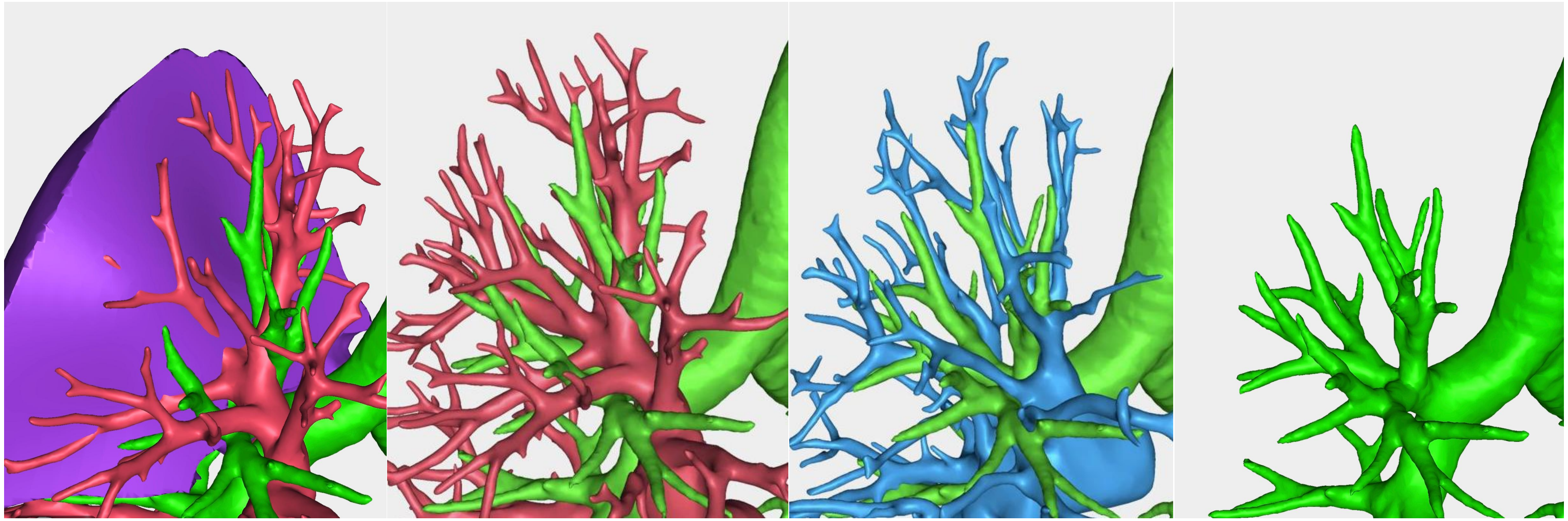

No. 3

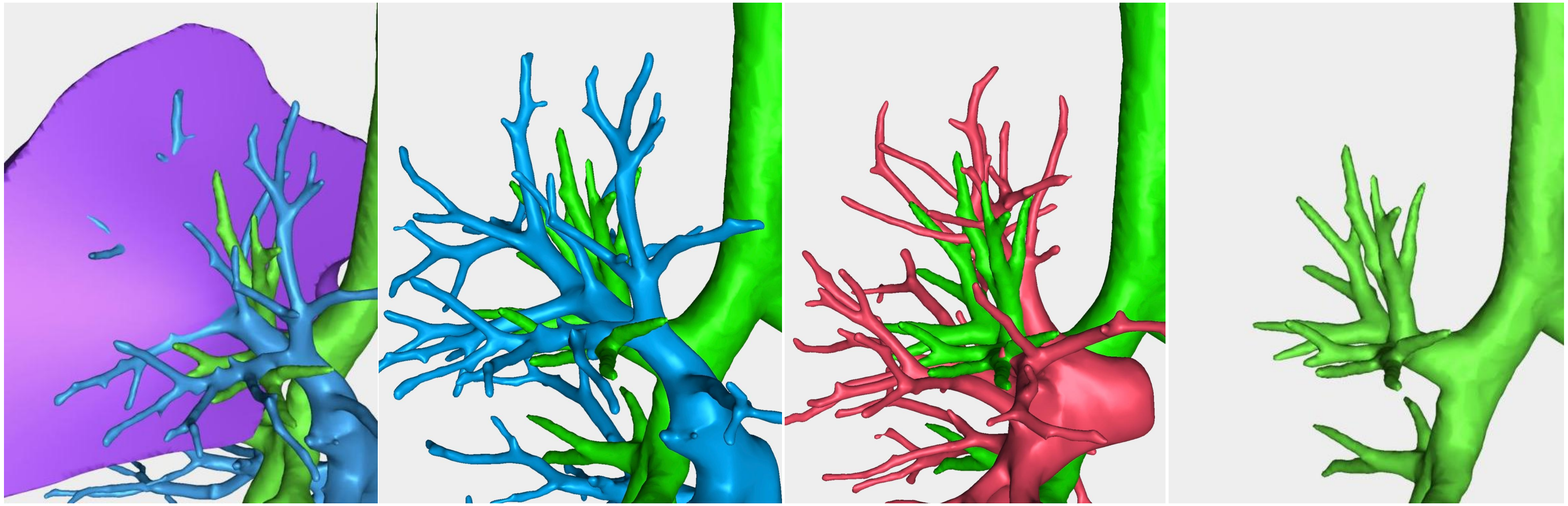

No. 4

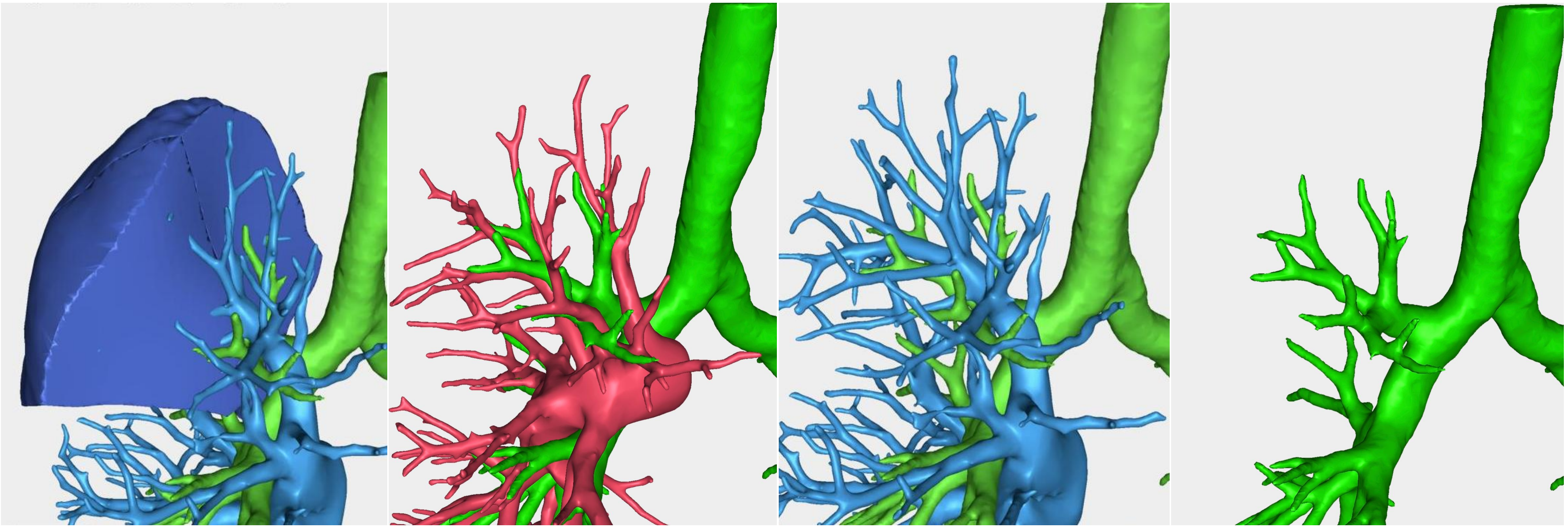

No. 5

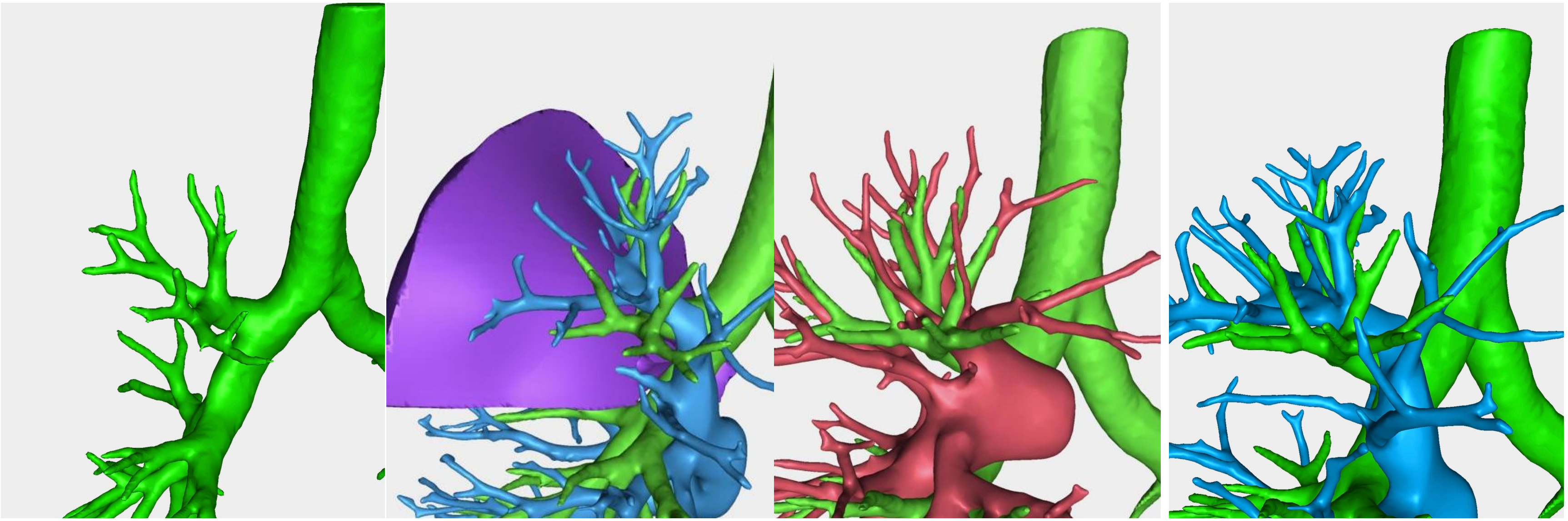

Supplement: Supplementary file 1 [file Datasheet1.pdf]
