# Supplementary material for: Concealed right upper lobe pulmonary venous variants on 3D-CTBA: morphological characterization and surgical implications
Source: Front Med (Lausanne). 2026 Sep 28;13:1964027. doi: 10.3389/fmed.2026.1964027 (PMC13655120; doi:10.3389/fmed.2026.1964027)

No.1

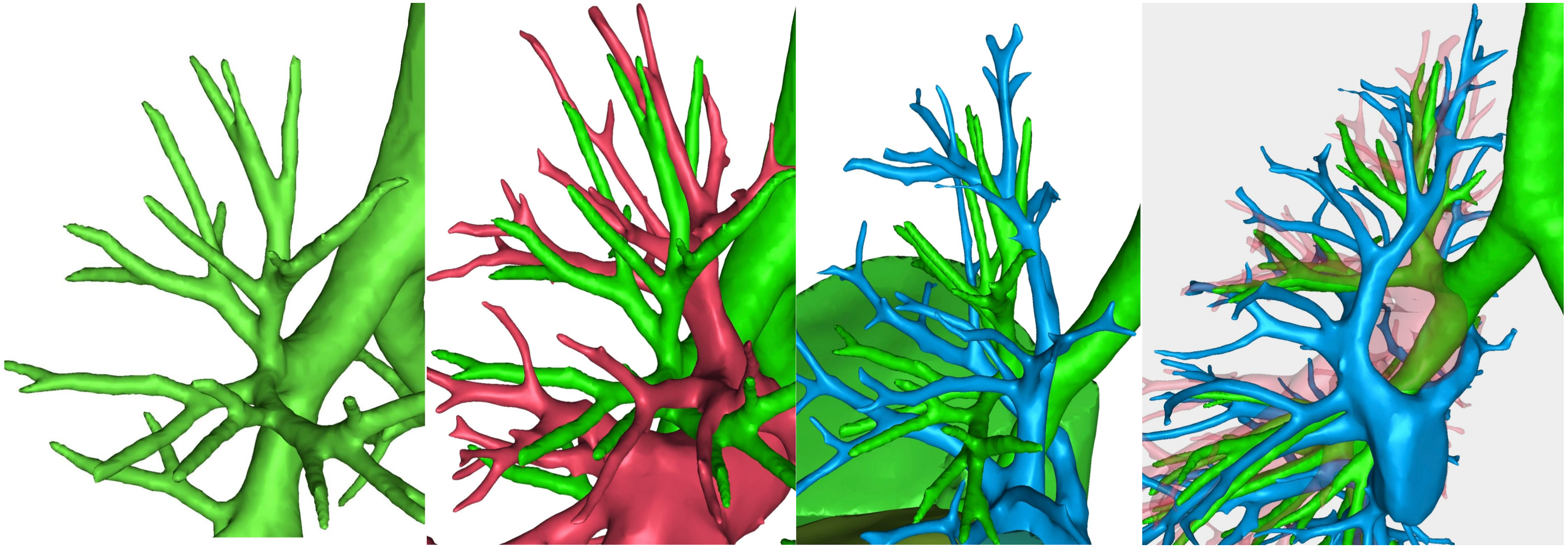

No.2

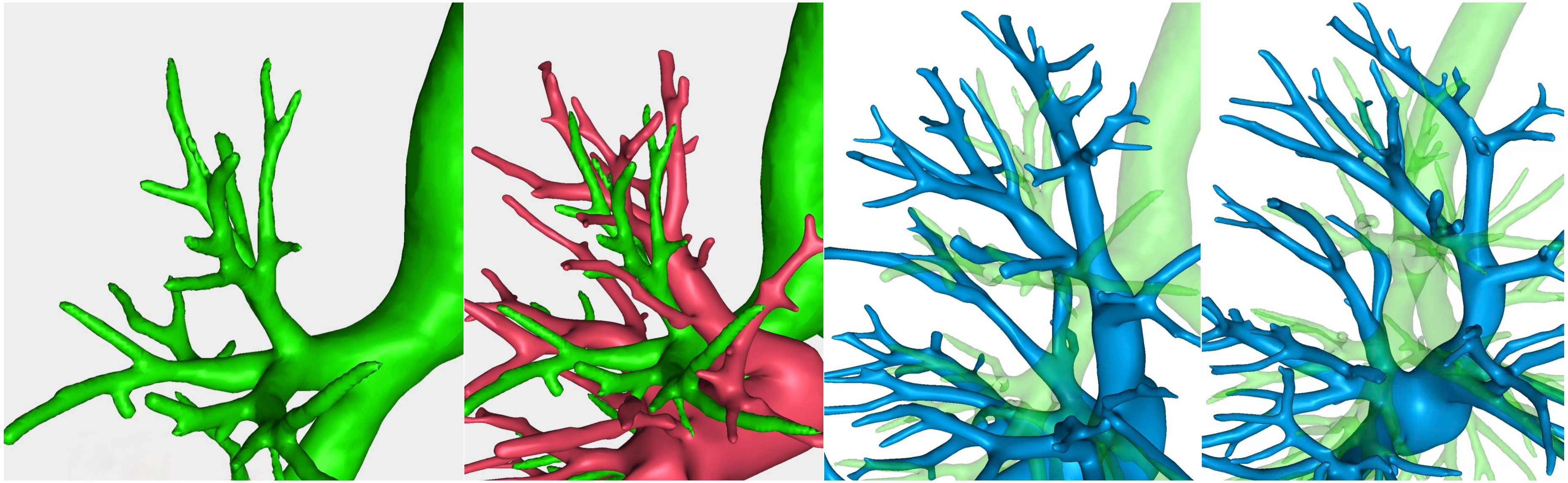

No.3

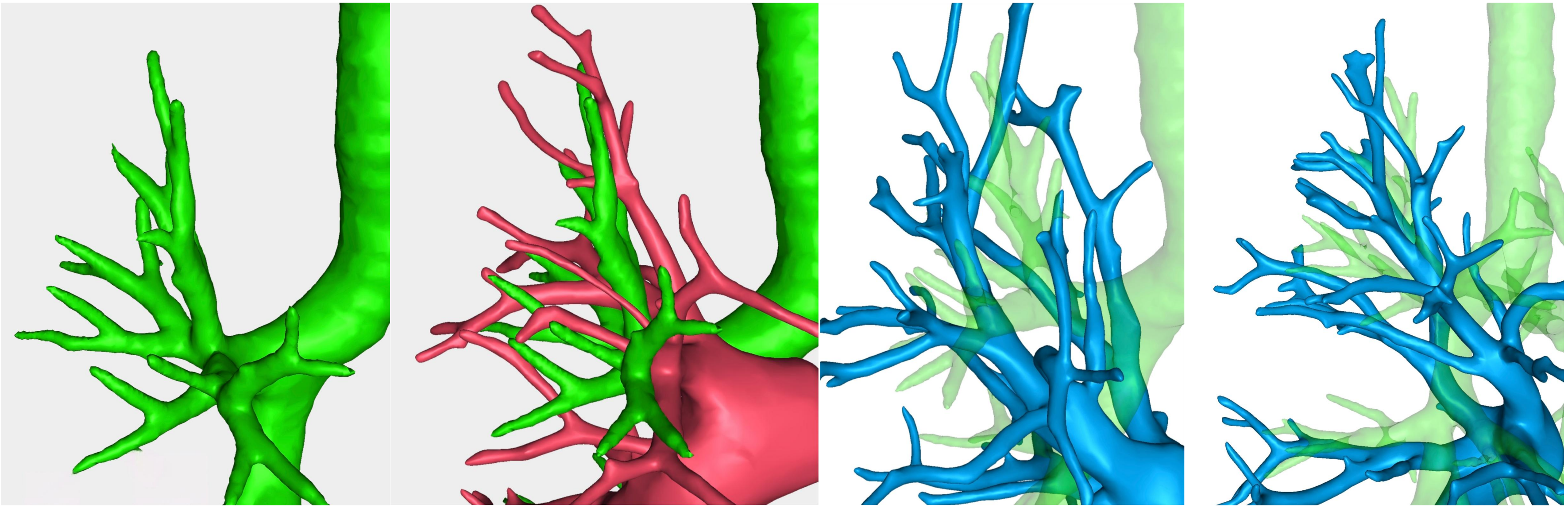

No.4

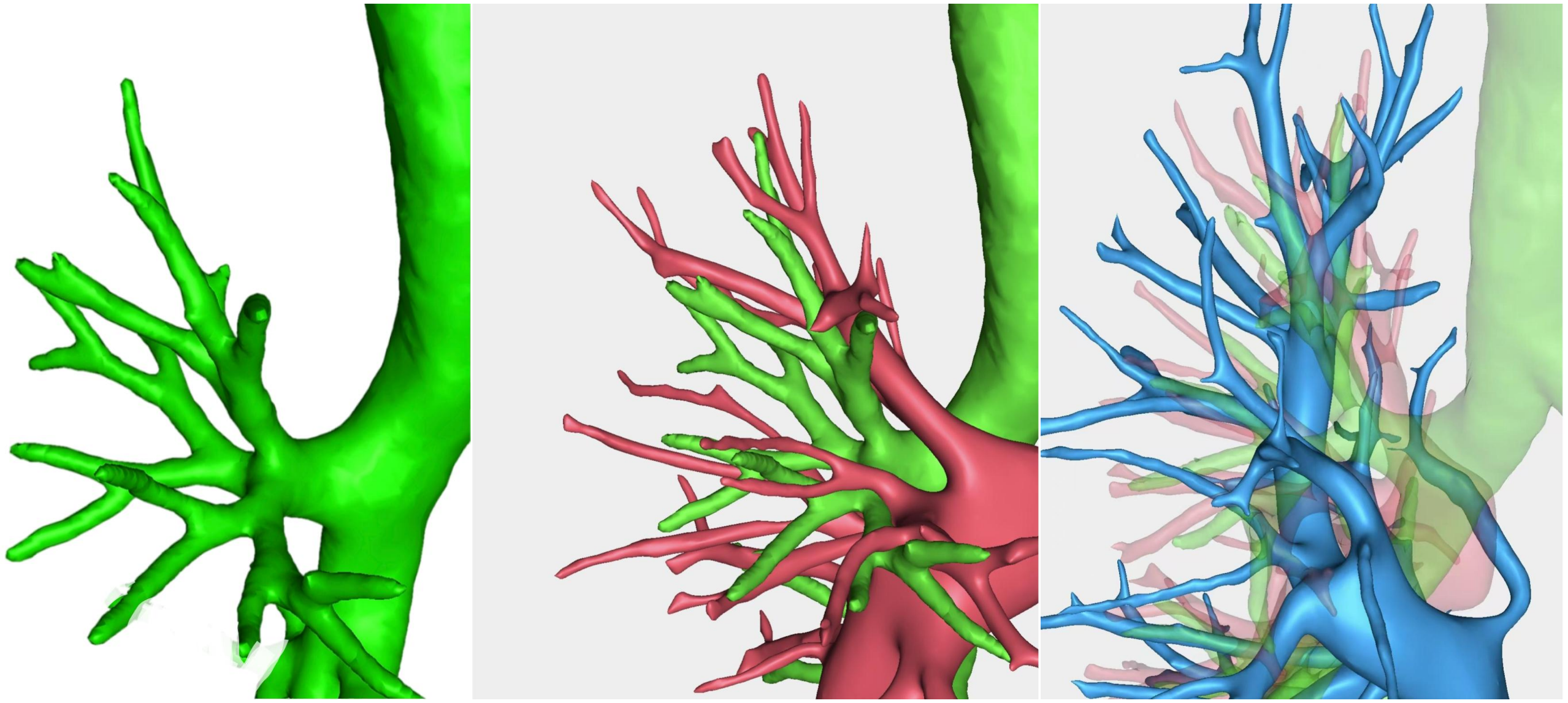

No.5

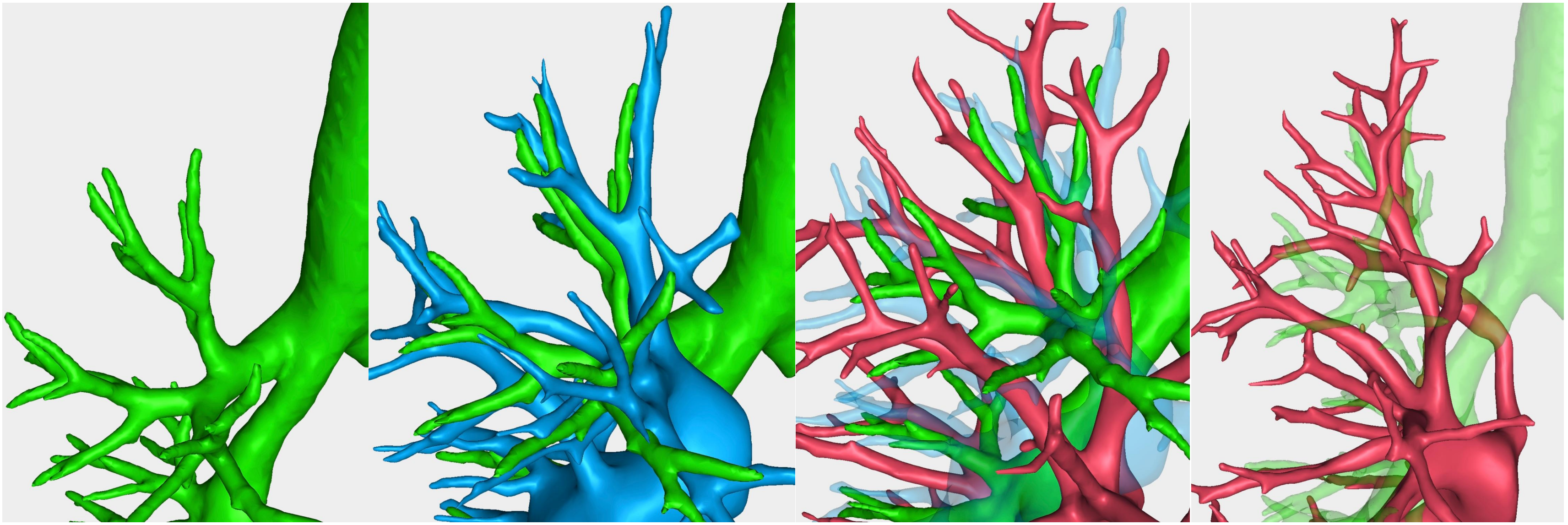

No.6

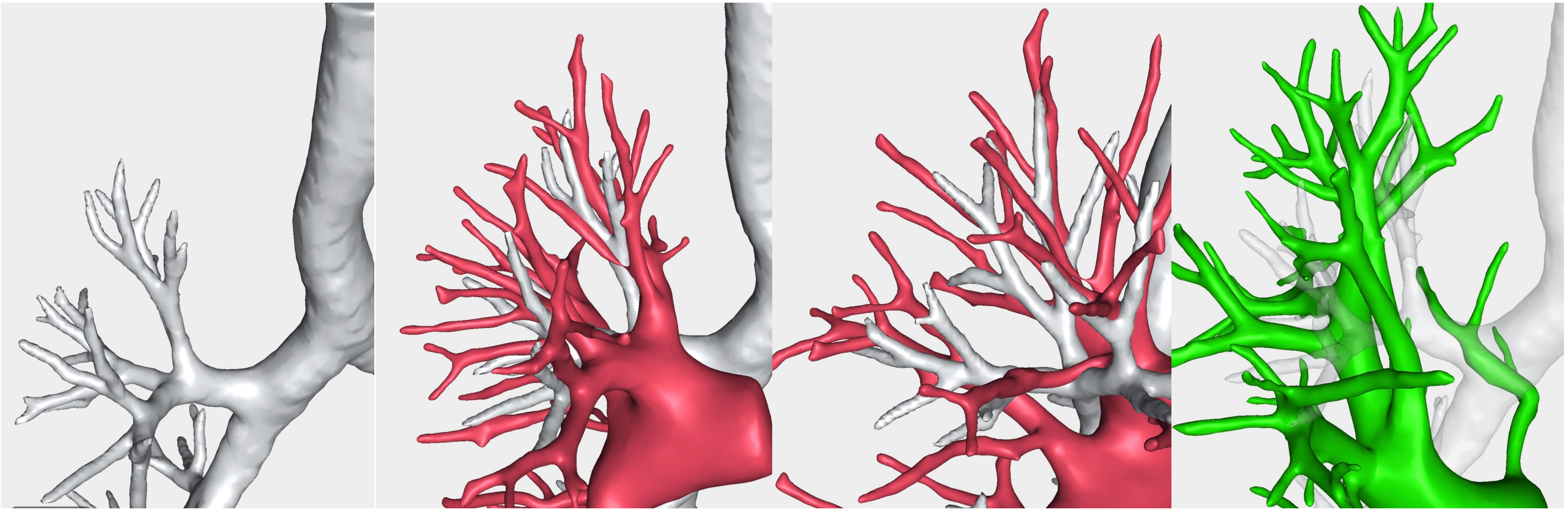

No.7

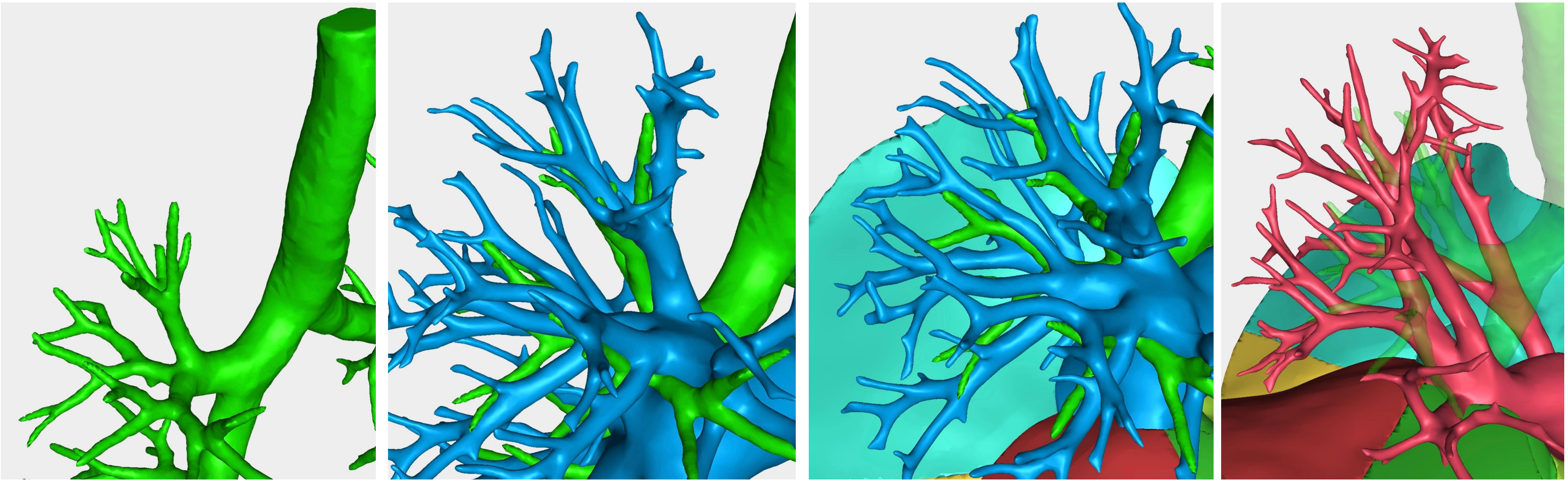

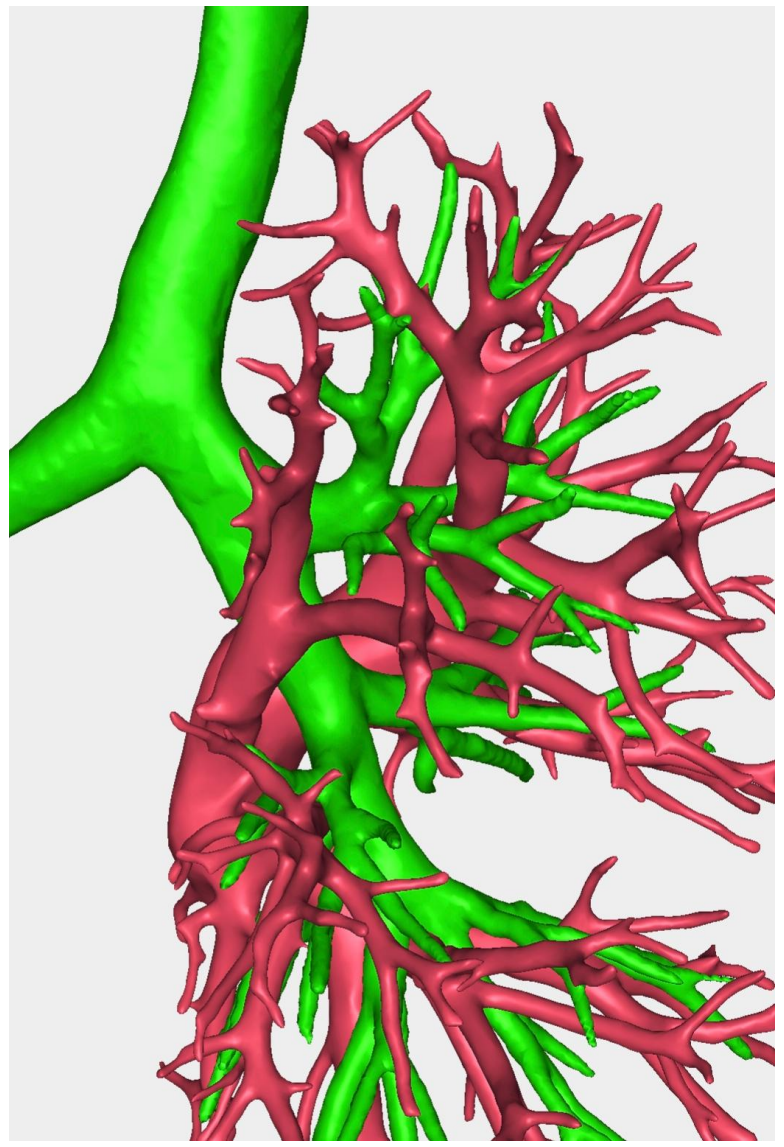

No.8

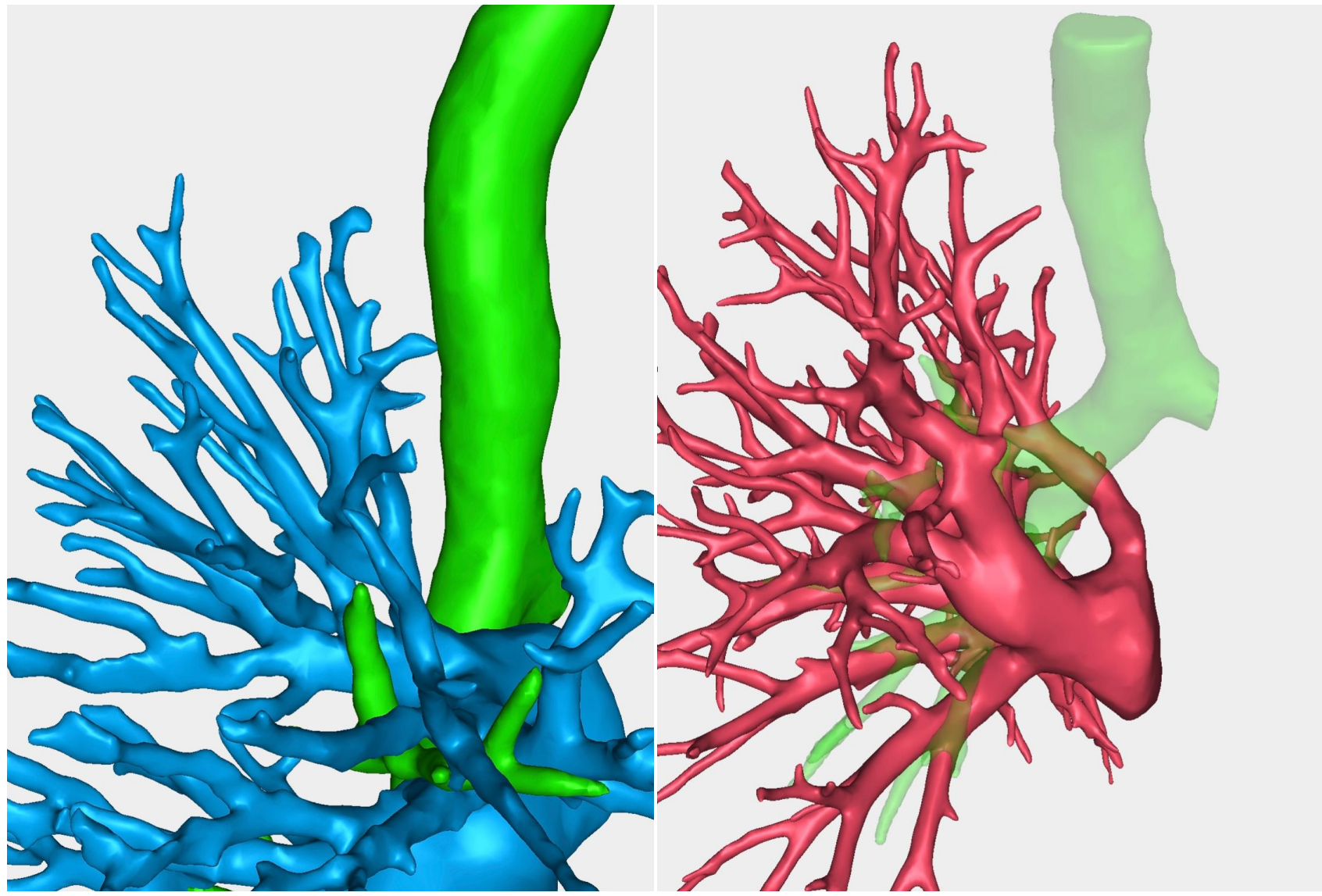

No. 9

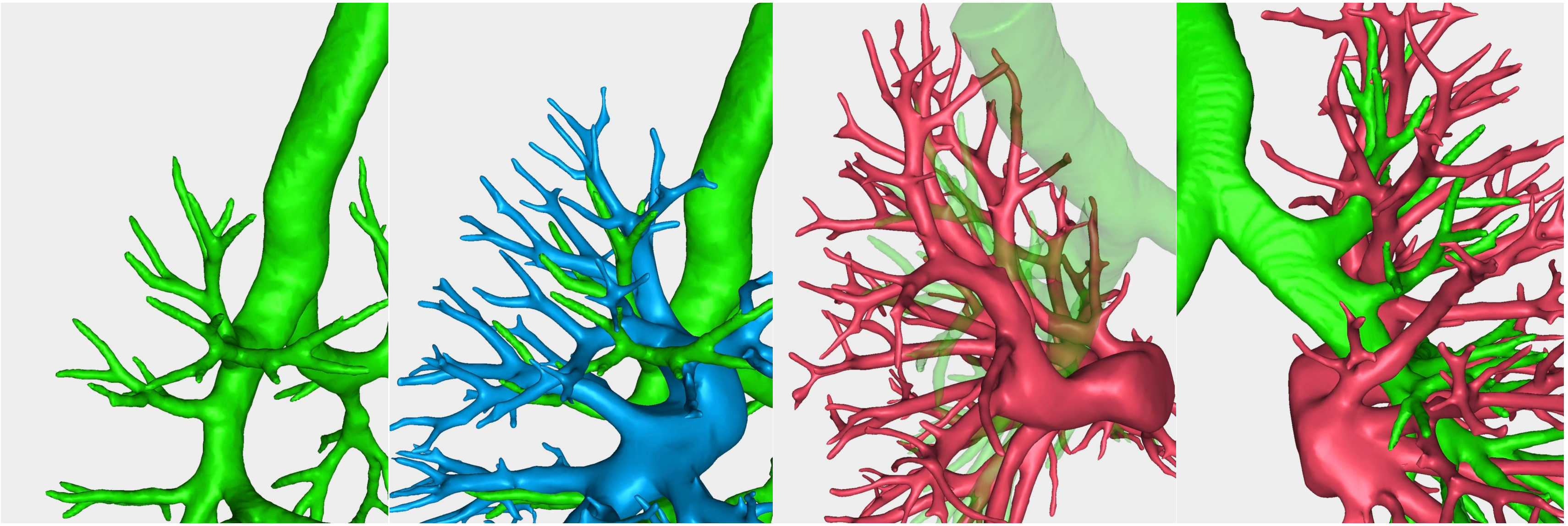

No. 10

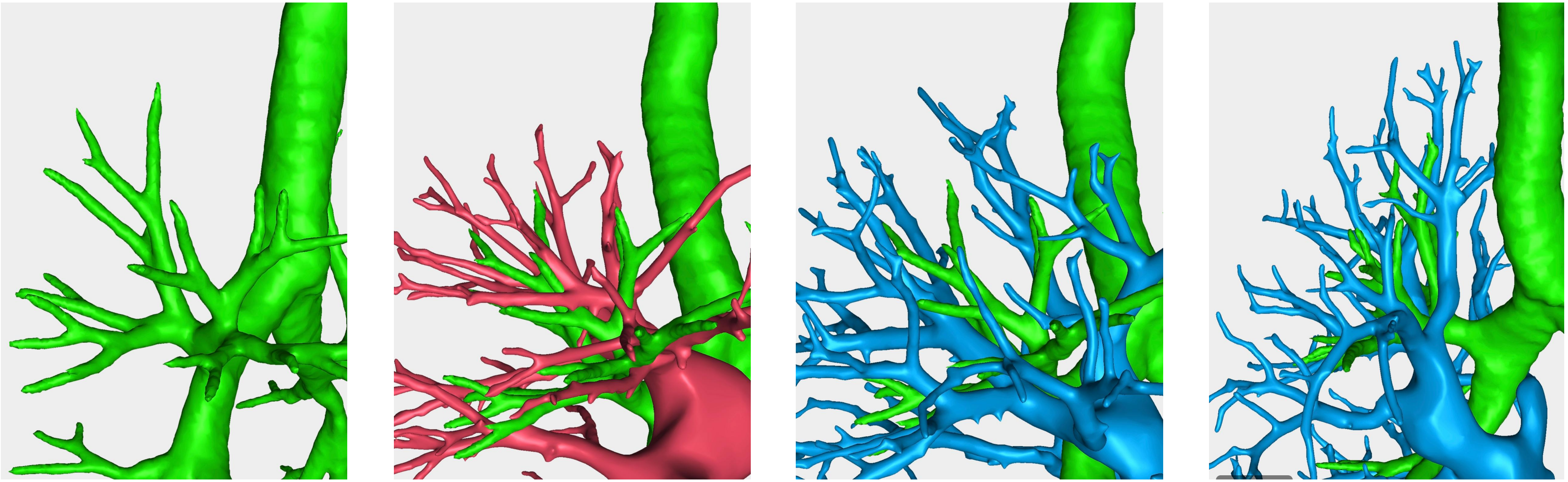

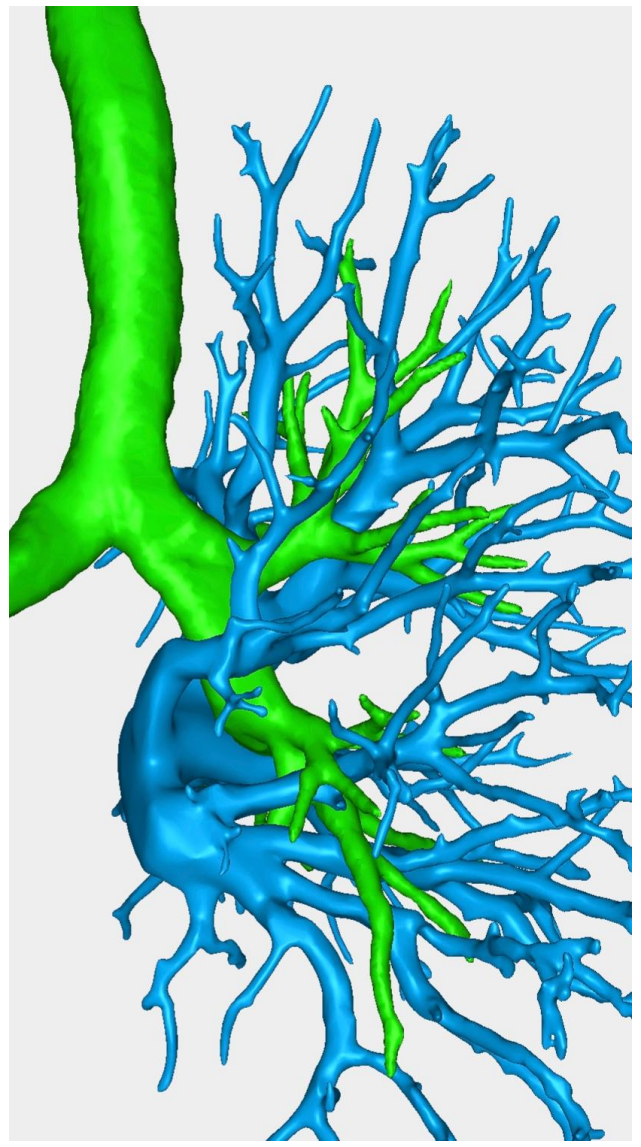

No. 11

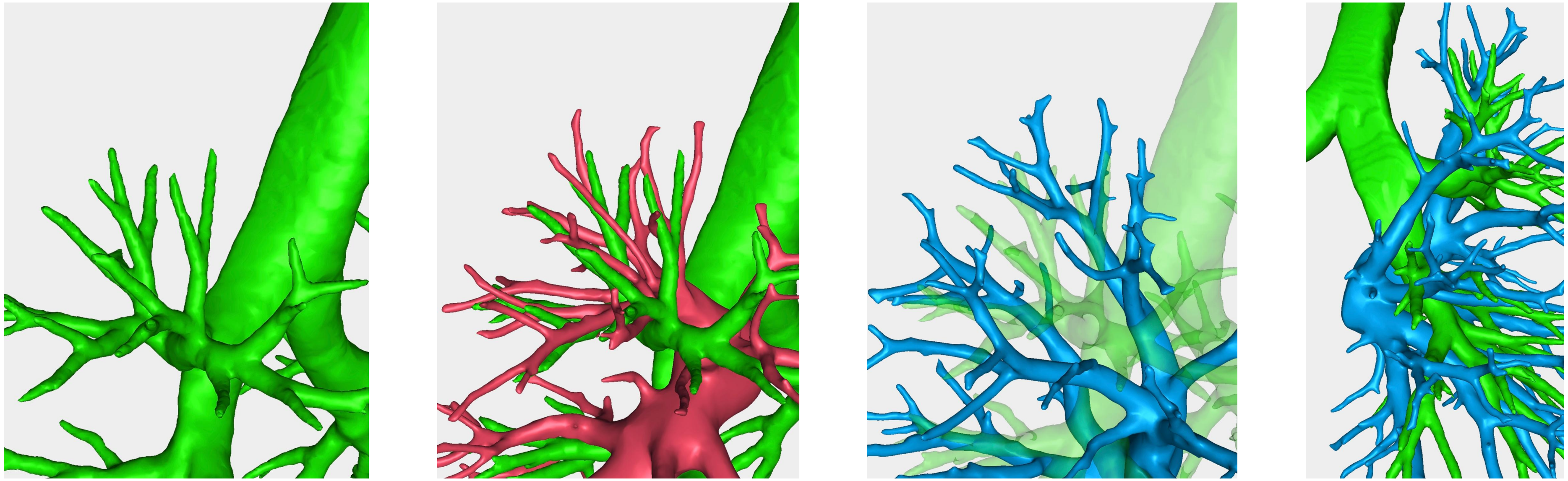

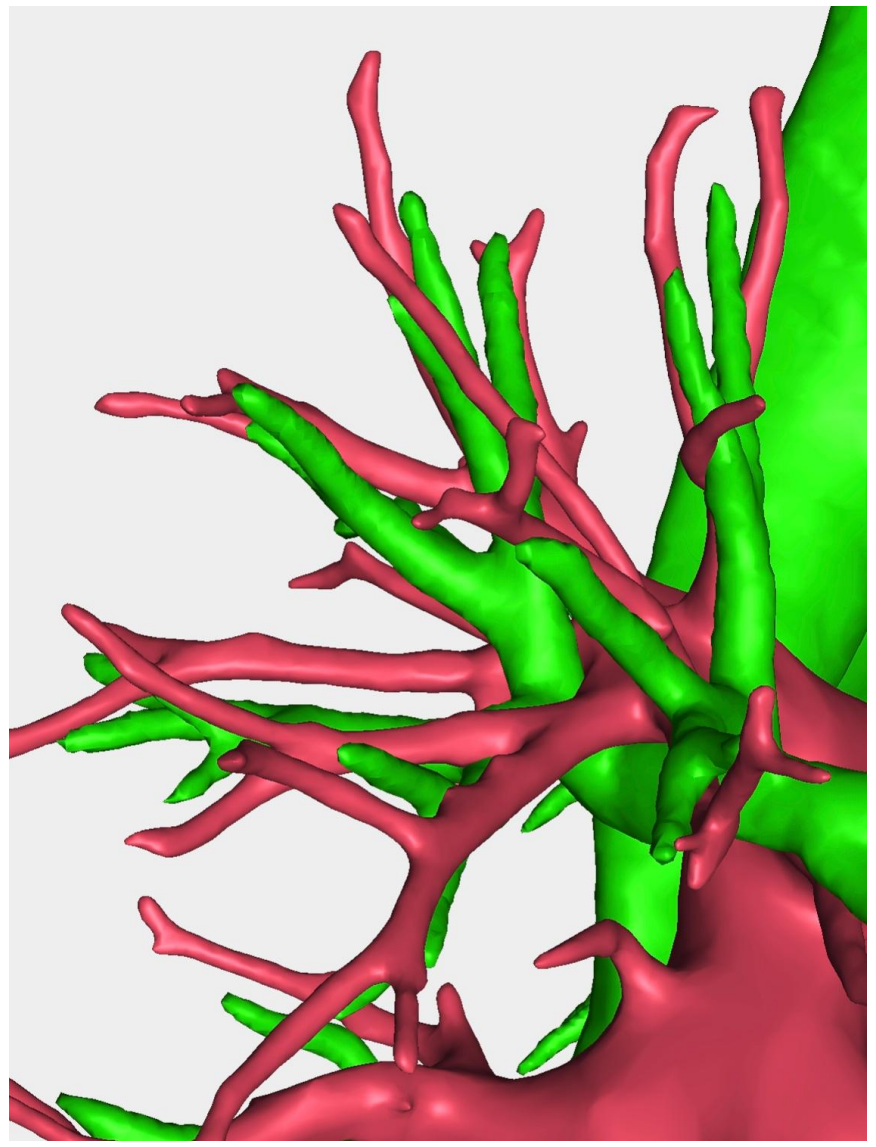

No.12

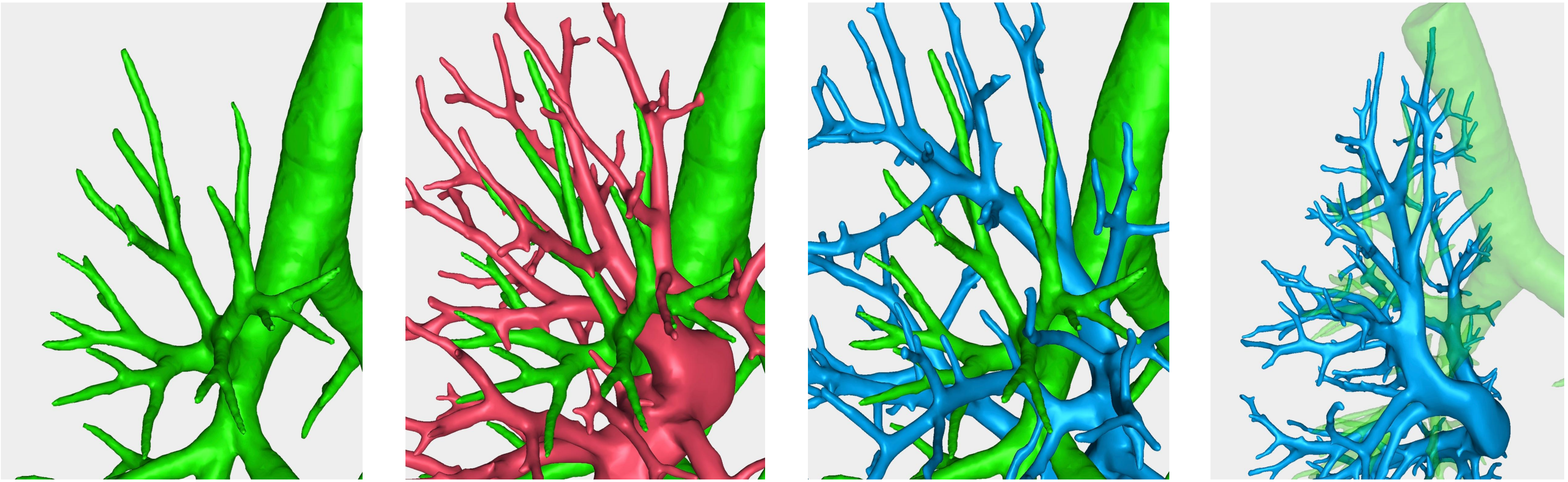

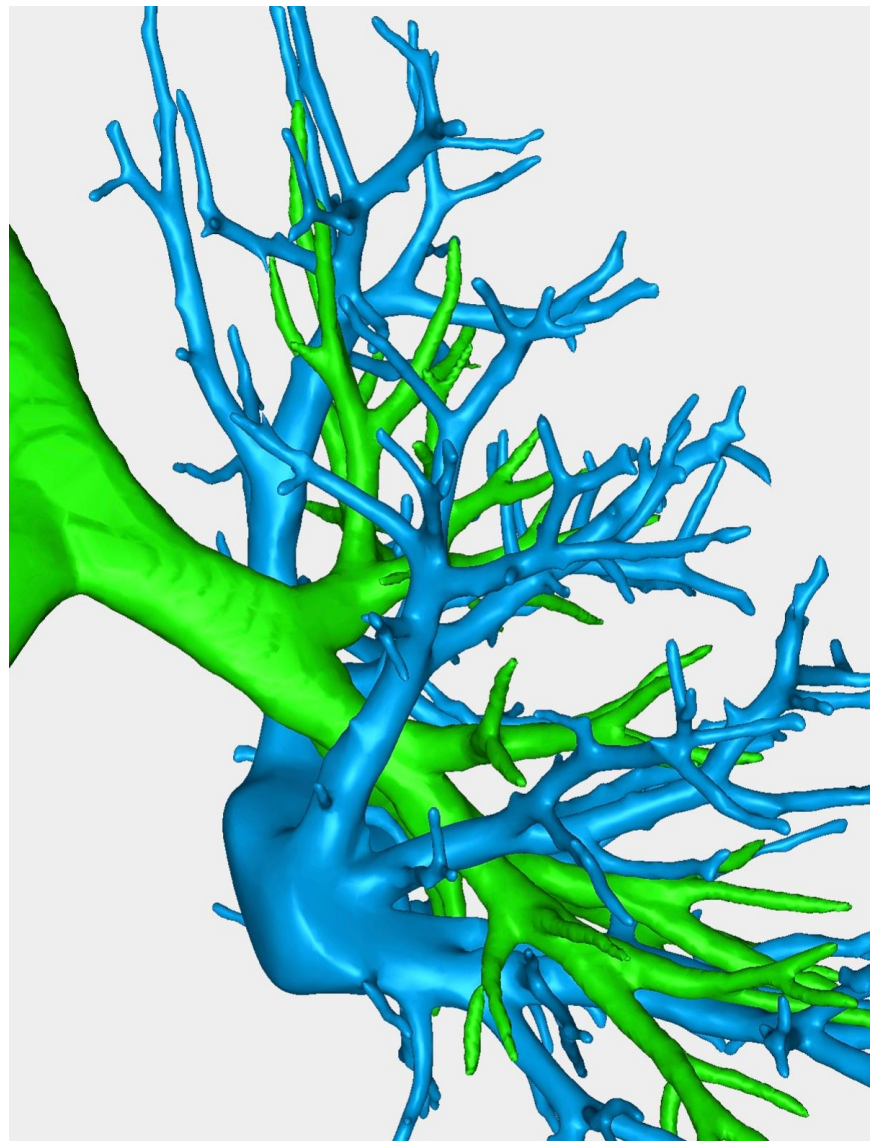

No. 13

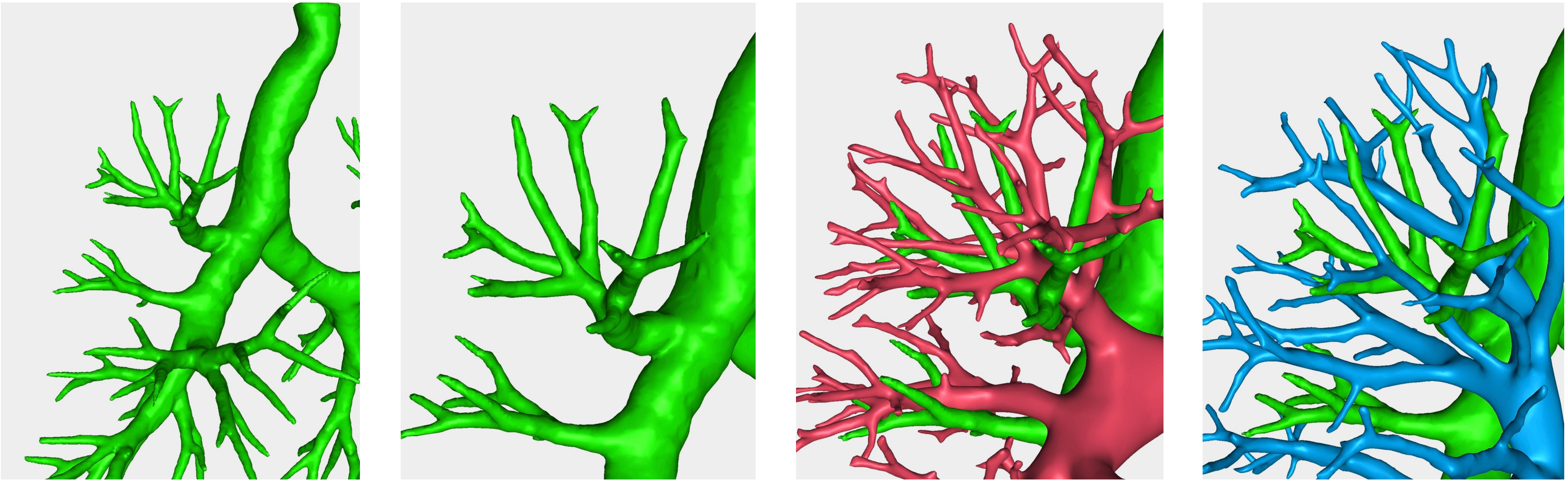

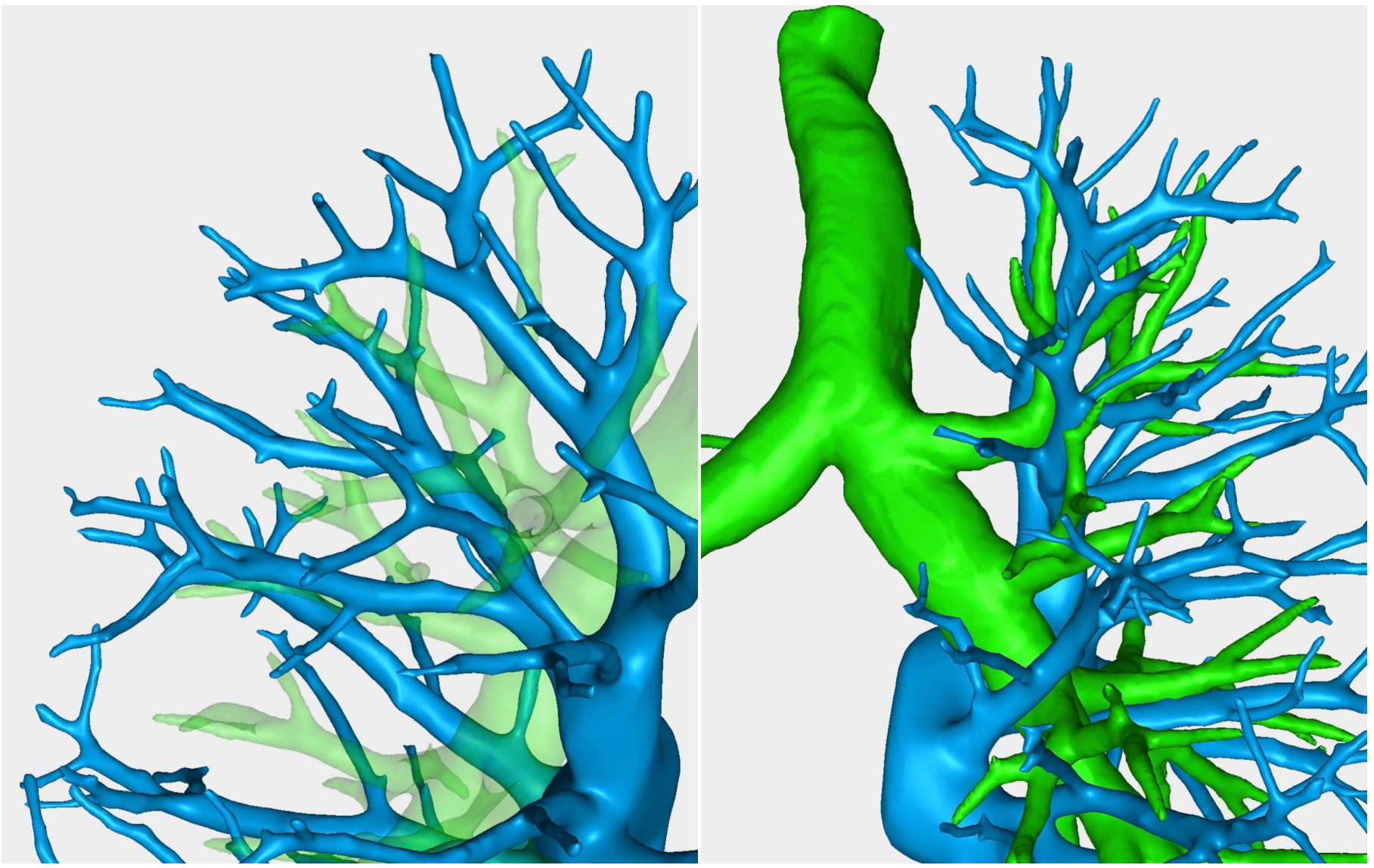

No. 14

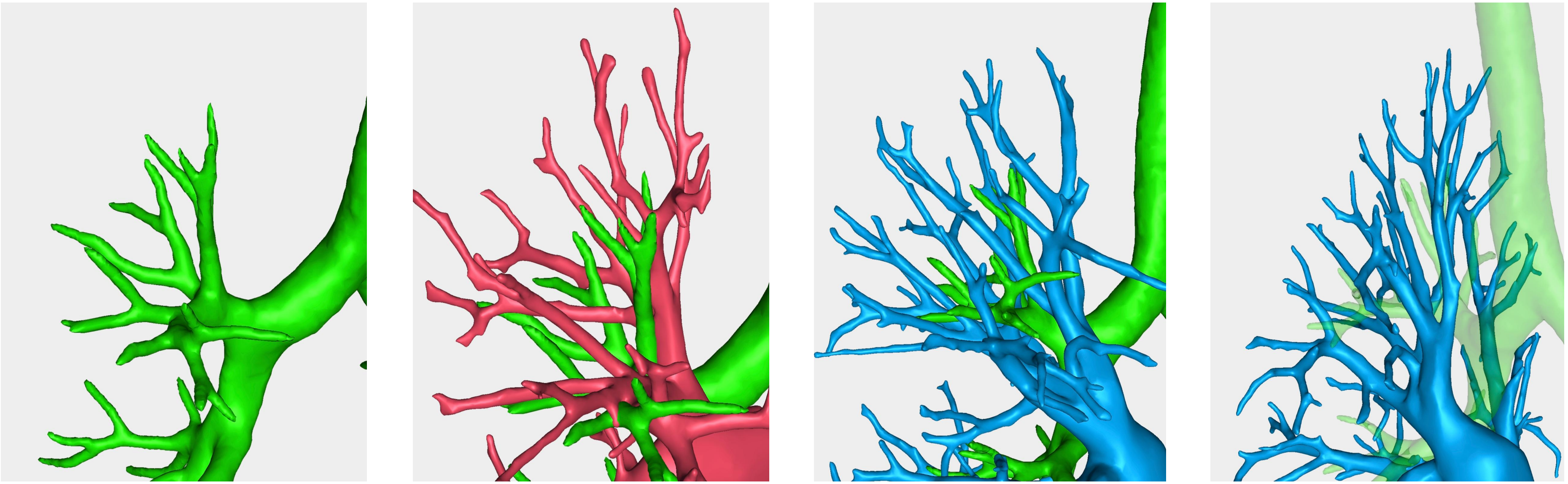

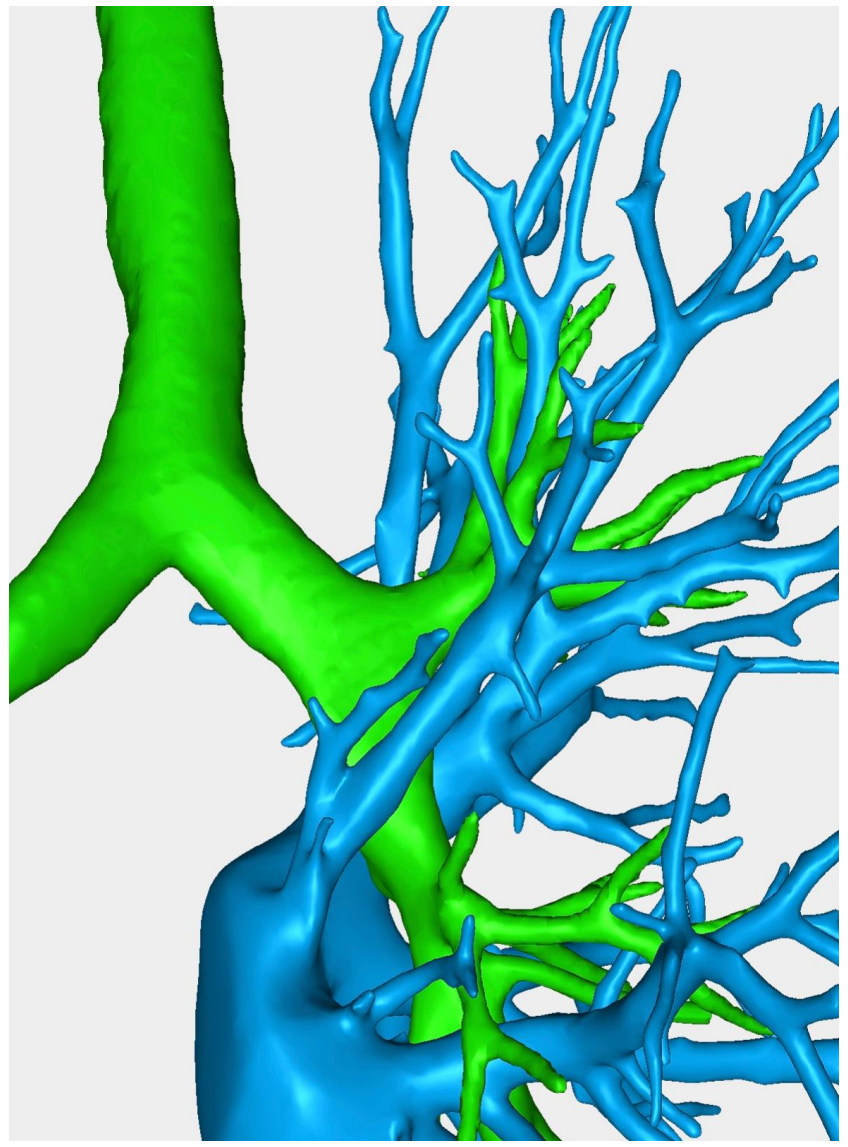

No. 15

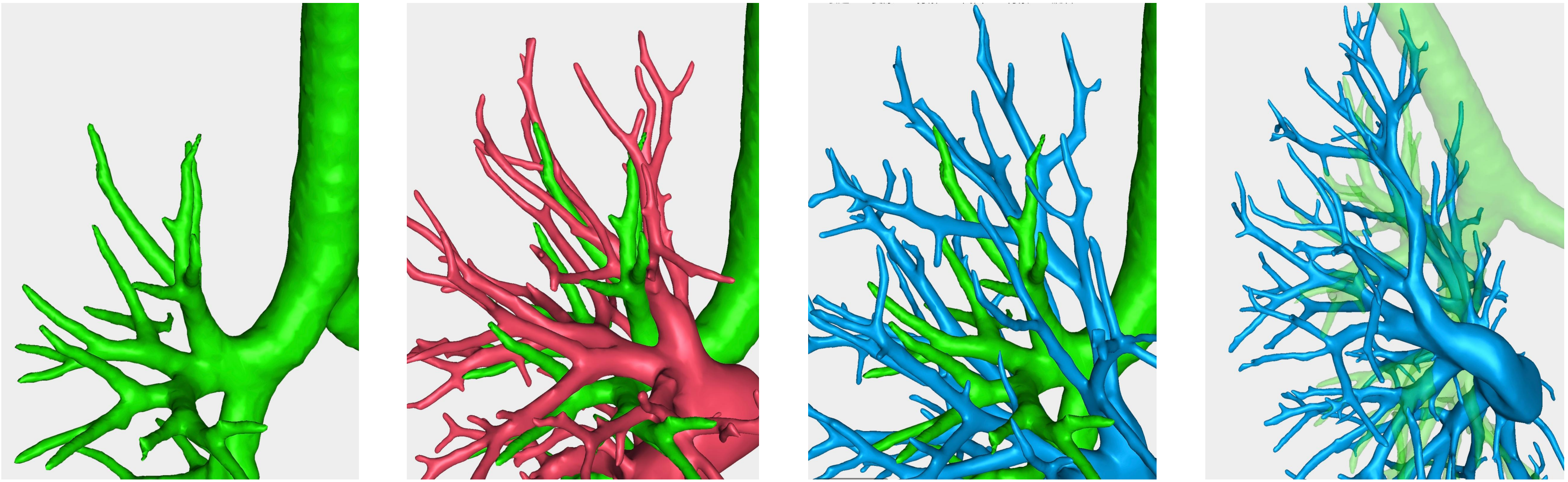

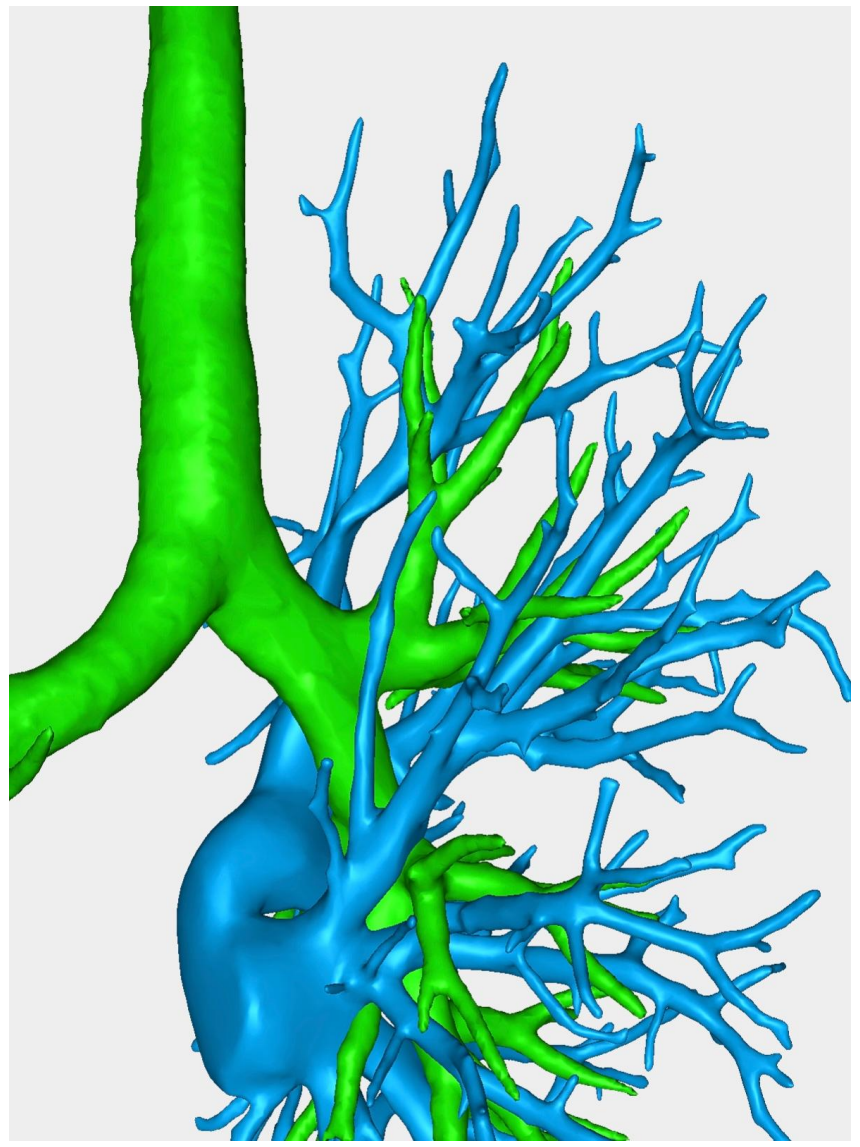

No. 16

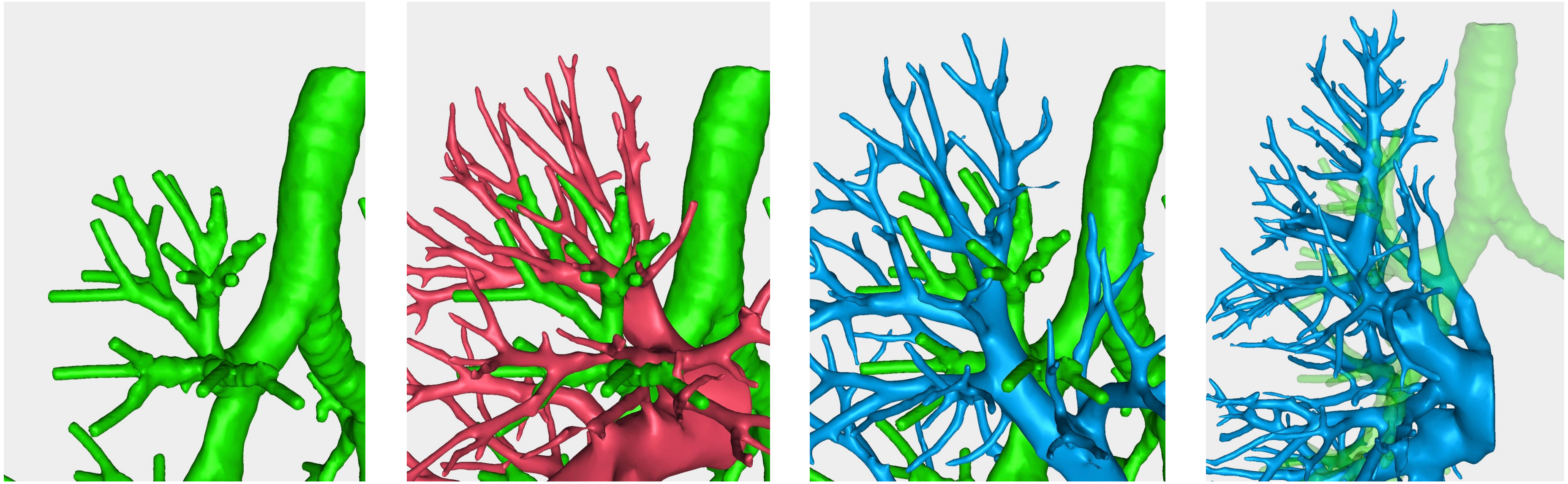

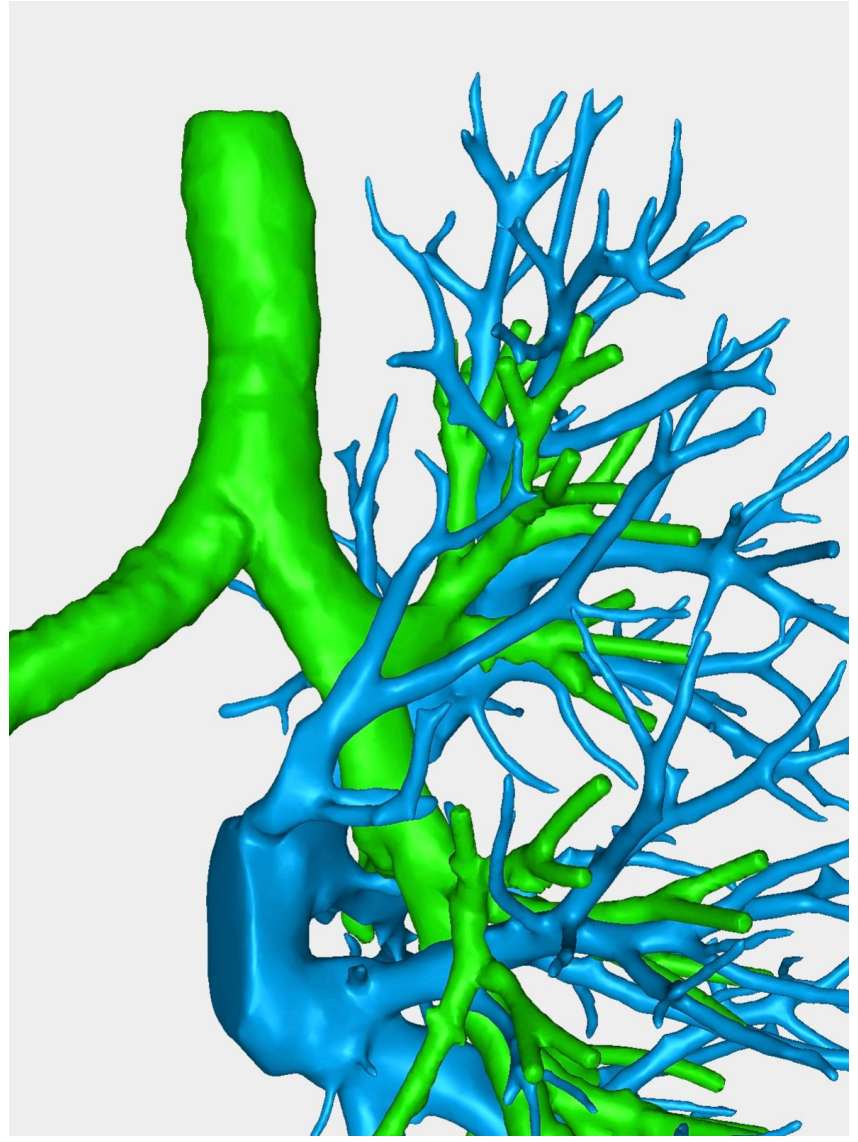

No. 17

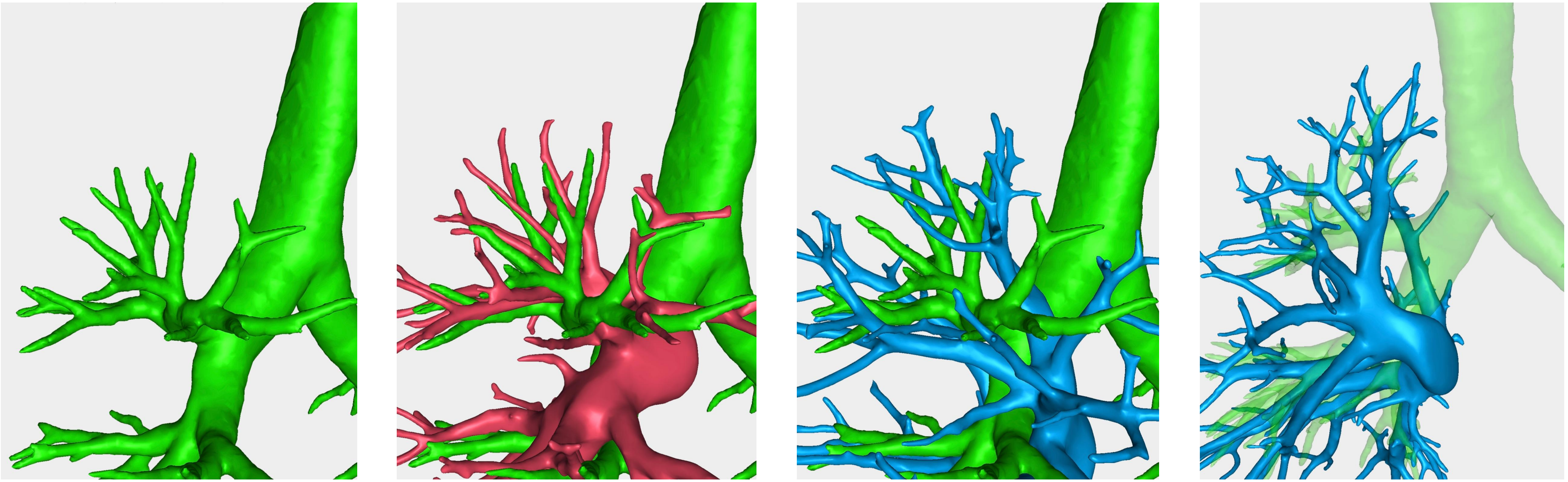

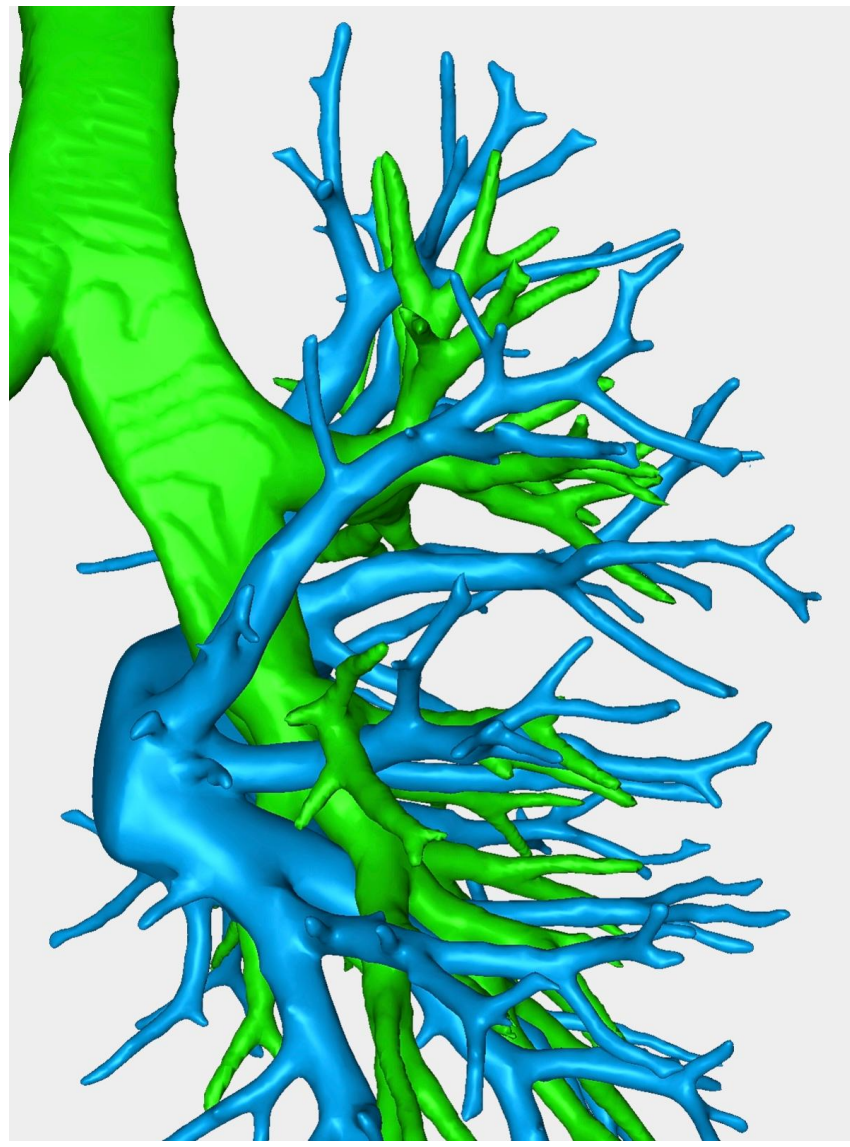

No. 18

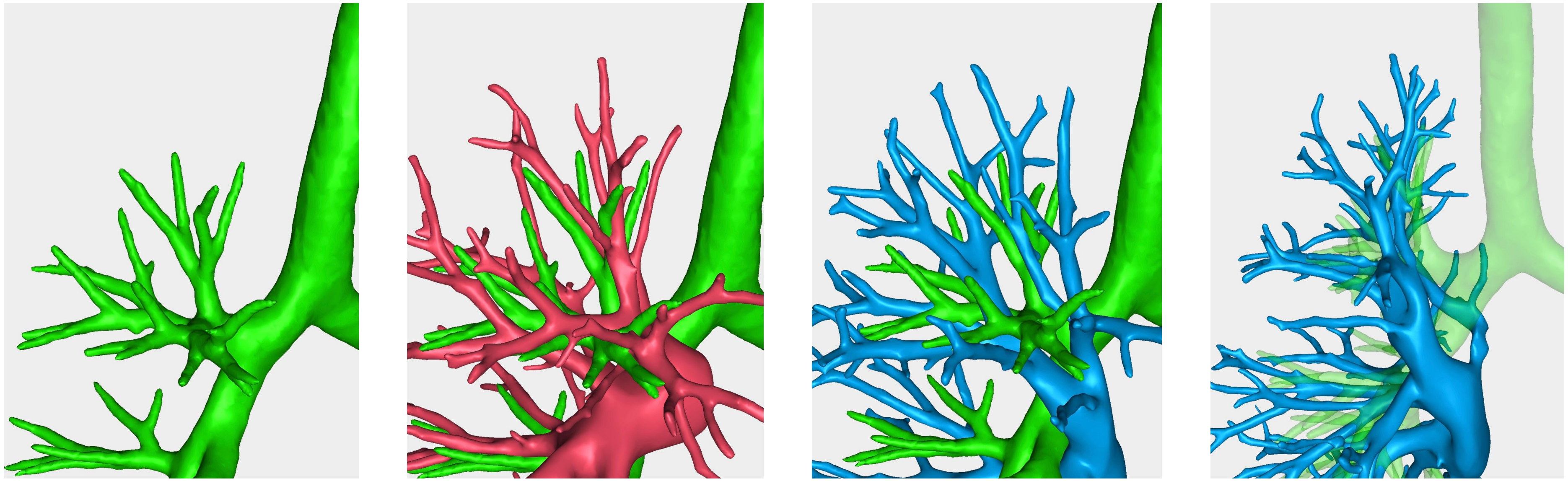

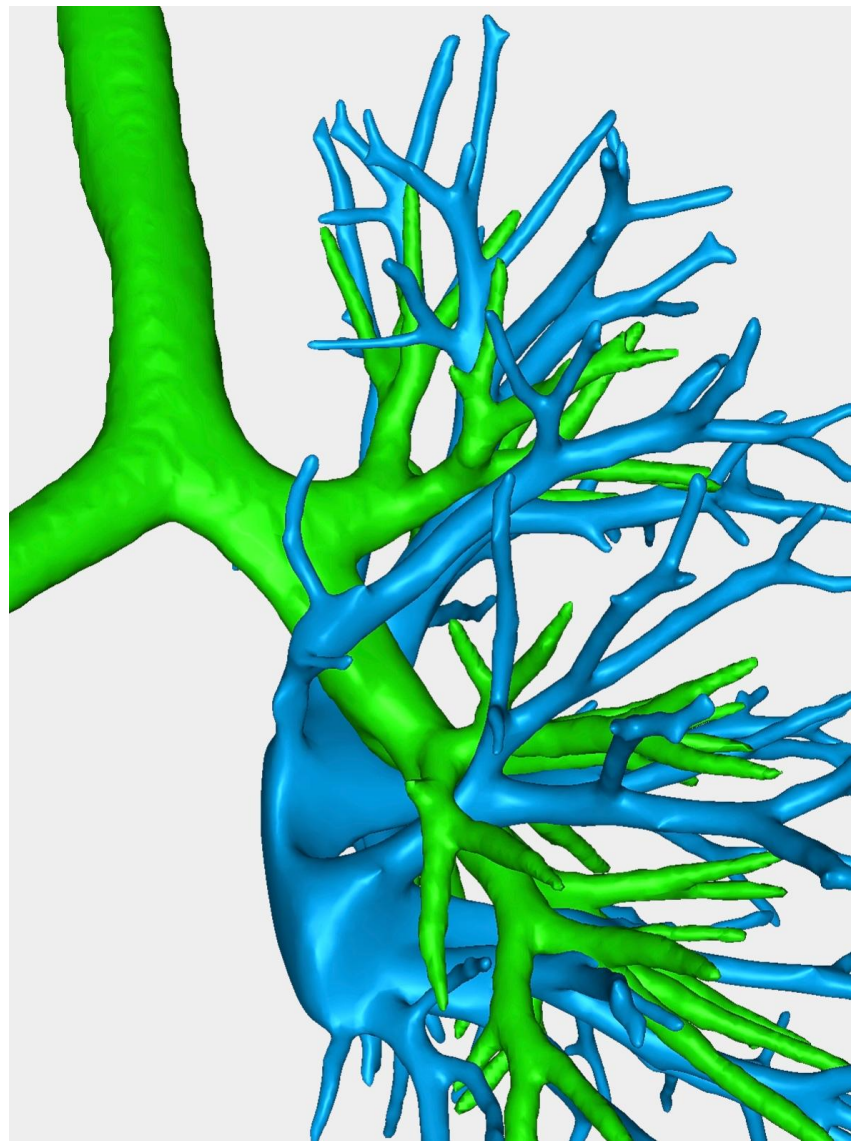

Supplement: Supplementary file 2 [file Datasheet2.pdf]
